# Injectable hydrogels for bone regeneration: from materials design to clinical translation

**DOI:** 10.1039/d6ra06569d

**Published:** 2026-09-01

**Authors:** Xinyue Zhang, Zheng Zhuang, Bo Liu, Mengjia Wu, Chenfei Lu, Zhan Zhang, Zilu Zhang, Xueyan Zhang, Yanguo Wang, Hao Hu

**Affiliations:** a College of Materials Science and Engineering, Qingdao University Qingdao 266071 China; b College of Materials Science and Engineering, Ocean University of China Qingdao 266100 China huhao@ouc.edu.cn; c Department of Orthopedics, Qilu Hospital (Qingdao), Cheeloo College of Medicine, Shandong University Qingdao 266035 China; d Marine Biomedical Research Institute of Qingdao Qingdao 266100 China

## Abstract

Clinical repair of bone defects has long faced challenges including the limited availability of autografts, immunological rejection of allografts, and mechanical mismatch of traditional implants. Injectable hydrogels have emerged as highly promising strategies in bone tissue engineering due to their unique advantages: minimally invasive implantation, the ability to conform to irregular defect cavities, excellent biocompatibility, and high functional tunability. This review systematically outlines recent advances in injectable hydrogels for bone regeneration. It comprehensively outlines material classifications and fundamental properties. Specifically, it analyzes key design strategies, including enhancing mechanical support through cross-linking optimization and the incorporation of reinforcing phases, reconstructing the regenerative microenvironment *via* bioactive factor loading, and enabling synchronized degradation and tissue regeneration. Furthermore, this review summarizes current applications for different bone defect types, identifies current technical bottlenecks such as balancing mechanical strength with minimally invasive delivery, and anticipates future directions, including stimuli-responsive design and multifunctional integration.

## Abstract 2

Clinical repair of bone defects has long faced challenges including the limited availability of autografts, immunological rejection of allografts, and mechanical mismatch of traditional implants.

## Graphical abstract

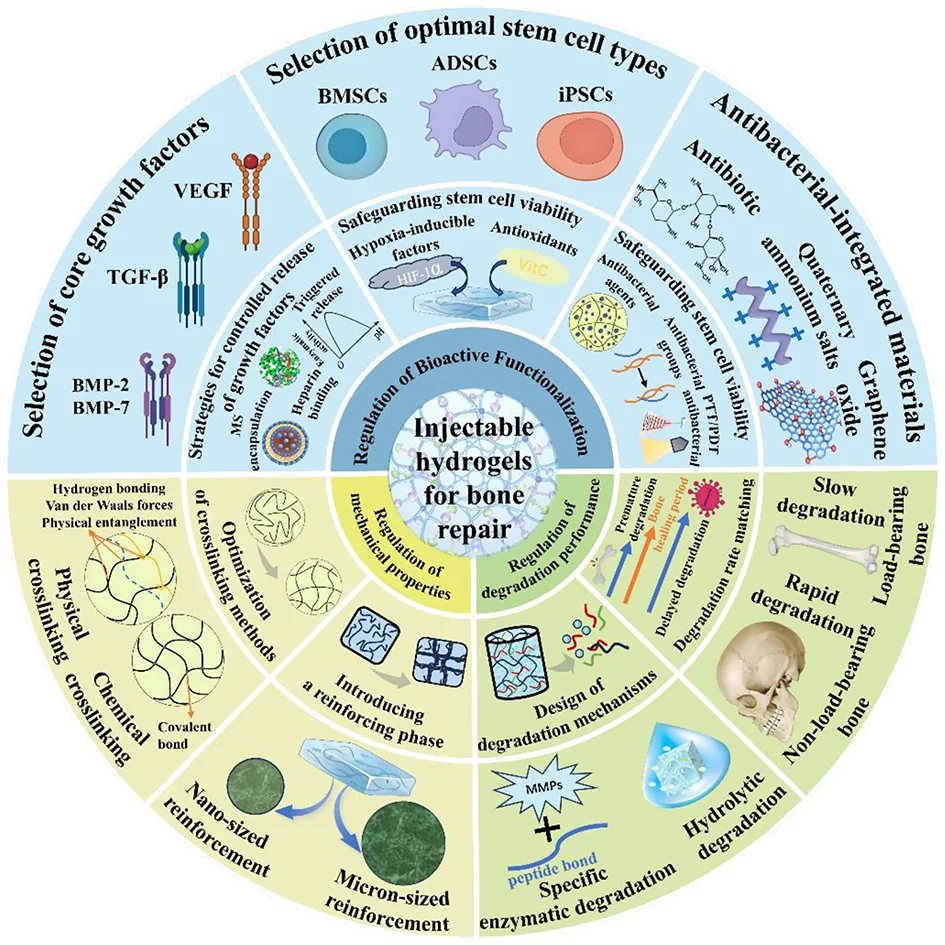

## 1. Introduction

Bones serve as the primary structural framework of the human body. They support body weight and protect internal organs. Furthermore, they form the structural basis of the musculoskeletal system, enabling complex movements. In clinical and daily settings, high-energy trauma, age-related bone loss, and various diseases can severely compromise bone integrity. Such damage severely restricts patients' motor function and may lead to long-term disability. Consequently, the efficient regeneration of damaged bone and restoration of its function remain major challenges in clinical practice.

Clinically, common bone defects include osteoporotic fractures, traumatic defects, post-tumoral defects, and other pathological defects. Osteoporotic fractures are among the most prevalent fracture types worldwide. Bone mass typically increases during childhood and adolescence, reaching a peak around the age of 30, after which it gradually decreases with advancing age. This decline significantly reduces bone strength and toughness, thereby increasing the risk of fractures even during minor trauma or routine activities. Research indicates that the incidence of these fractures increases annually among the global population aged over 50. Hip fractures exhibit the highest mortality and disability rates, and patients rarely regain their pre-injury functional status.^1^ This incidence closely correlates with population aging; approximately one-third of women and one-fifth of men worldwide will experience an osteoporotic fracture in their lifetime.^2^ Traumatic bone defects present a significant challenge across all age groups. These defects frequently accompany soft tissue injuries and vascular or nerve damage. Approximately 5 to 10% of such fracture cases progress to delayed healing or non-union.^3^ Severe cases require multiple bone grafting surgeries and even carry a risk of amputation. Bone defects are an inevitable complication following tumor treatment. This outcome primarily results from the extensive resection required during tumor surgery. Bone tumor patients are predominantly young or middle-aged adults who have high demands for limb function recovery. However, postoperative defect areas often suffer from impaired blood supply and disrupted bone metabolism. Consequently, these patients frequently require secondary or even multiple reconstructive surgeries. Besides tumors, factors like bone infections and congenital bone deformities can cause pathological bone defects. Although these cases account for a small proportion, the healing process remains extremely difficult.^4^

Current clinical bone repair strategies have inherent limitations that restrict repair outcomes and patient prognosis. Autologous bone grafting offers excellent conductivity, inductivity, and biocompatibility. However, it is limited by the availability of donor bone. The limited harvest volume cannot meet the demands of repairing large-area defects.^5^ Furthermore, the donor site is susceptible to complications such as pain and infection, thereby causing secondary trauma to patients.^6,7^ Allogeneic bone grafting alleviates donor scarcity but introduces risks of immune rejection and potential disease transmission. During the healing phase, these grafts exhibit low osteoinductive activity, resulting in relatively long healing periods.^8^ Metal implants primarily provide fracture fixation and mechanical support. Although titanium and its alloys are widely used clinically due to their excellent mechanical properties and biocompatibility, they are bioinert and do not actively stimulate new bone formation compared to bioactive materials. Long-term retention in the body triggers inflammatory responses, leading to loosening or displacement. Most patients require a secondary surgery for removal, which increases the risk of infection.^9,10^ Traditional bone cements cure rapidly and provide mechanical support. Nevertheless, their mechanical properties rarely match those of natural bone tissue. This mismatch causes stress shielding and surrounding bone resorption, ultimately prolonging recovery time. If bone cement leaks into the venous system, it can cause cardiopulmonary embolism and other complications.^11^ Additionally, bone cements are non-degradable. Long-term retention can lead to aging and fragmentation, which impair limb function. These limitations make it difficult for traditional strategies to balance repair efficacy, patient comfort, and long-term safety.^12^ Traditional approaches particularly struggle to meet the diverse clinical demands for precise treatment of irregular, complex traumatic and pathological bone defects.

Addressing these limitations requires a paradigm shift from rigid, pre-formed implants to adaptable biomaterials compatible with minimally invasive procedures. Unlike conventional grafts that often necessitate extensive surgical exposure, injectable hydrogels offer a unique solution by transitioning from a fluid to a solid state *in situ*. This distinct characteristic not simply circumvents the trauma associated with open surgery but also ensures precise conformity to irregular defect geometries, thereby establishing a critical foundation for the advanced functional advantages detailed in the following section.

**Fig. 1:**
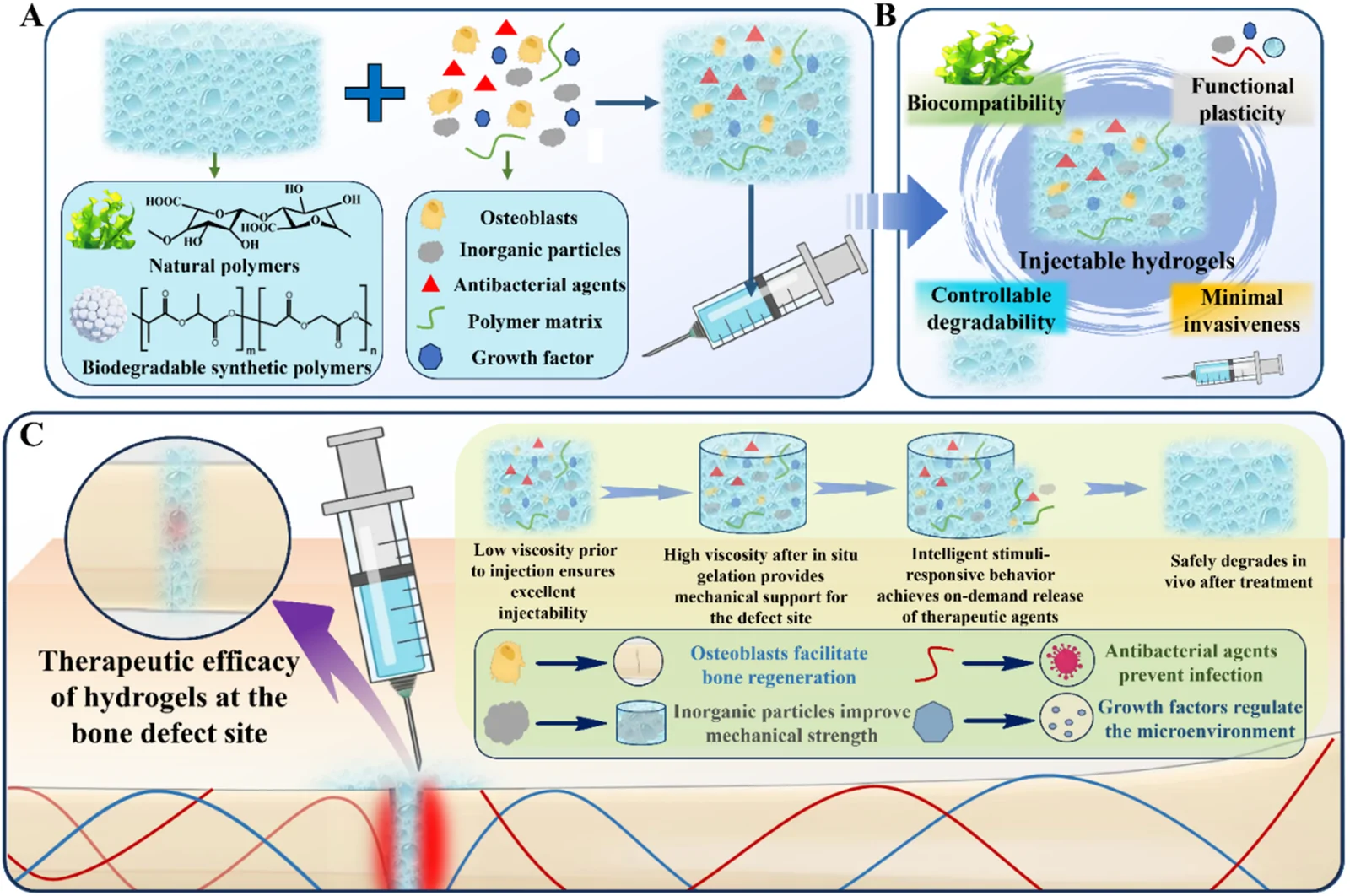
Strategy for injectable hydrogel for bone repair. (A) Schematic illustration of the hydrogel's structural design. (B) Key advantages of the hydrogel for bone regeneration. (C) Schematic of the therapeutic process.

Driven by the clinical demand for minimally invasive procedures, injectable hydrogels have emerged as a superior alternative to conventional grafts. Injectable hydrogels are hydrophilic polymer materials featuring a three-dimensional (3D) network structure.^13,14^ Unlike rigid scaffolds that struggle to fit complex geometries, injectable hydrogels can be delivered through small incisions as precursors, flowing into and perfectly conforming to irregular bone defects to ensure intimate tissue integration.^15,16^ These operational benefits are fundamentally supported by superior rheological tunability. Specifically, the optimized viscosity allows for manageable injection forces, while the inherent shear-thinning behaviour significantly reduces flow resistance during extrusion, facilitating smooth delivery through fine needles. Crucially, the rapid thixotropic recovery ensures immediate structural stabilization upon deposition, effectively preventing post-injection leakage and maintaining defect-filling fidelity. Furthermore, their composition can be tailored to possess excellent biocompatibility, which significantly reduces the risk of immune rejection and inflammatory responses often triggered by foreign implants.^17,18^ Crucially, these materials offer a multifunctional platform that extends beyond simple defect filling. They can be engineered to load and sustain the release of multiple osteogenic factors and encapsulate therapeutic cells, thereby creating a bioactive niche that actively accelerates regeneration (Fig. 1).^19–21^ By combining the surgical benefits of minimal invasiveness with these versatile biological functions, injectable hydrogels provide a comprehensive solution for efficient bone repair.^22^

This review summarizes the research and development progress of injectable hydrogels from material foundations to clinical translation. Specifically, it systematically outlines material classifications and design principles, details the mechanisms underlying bone repair, and highlights recent advances in functional modification and applications. Finally, the review discusses pivotal challenges and future directions in the development of ideal injectable hydrogels for bone repair.

## 2. Key design strategies of injectable hydrogels for bone regeneration

**Fig. 2:**
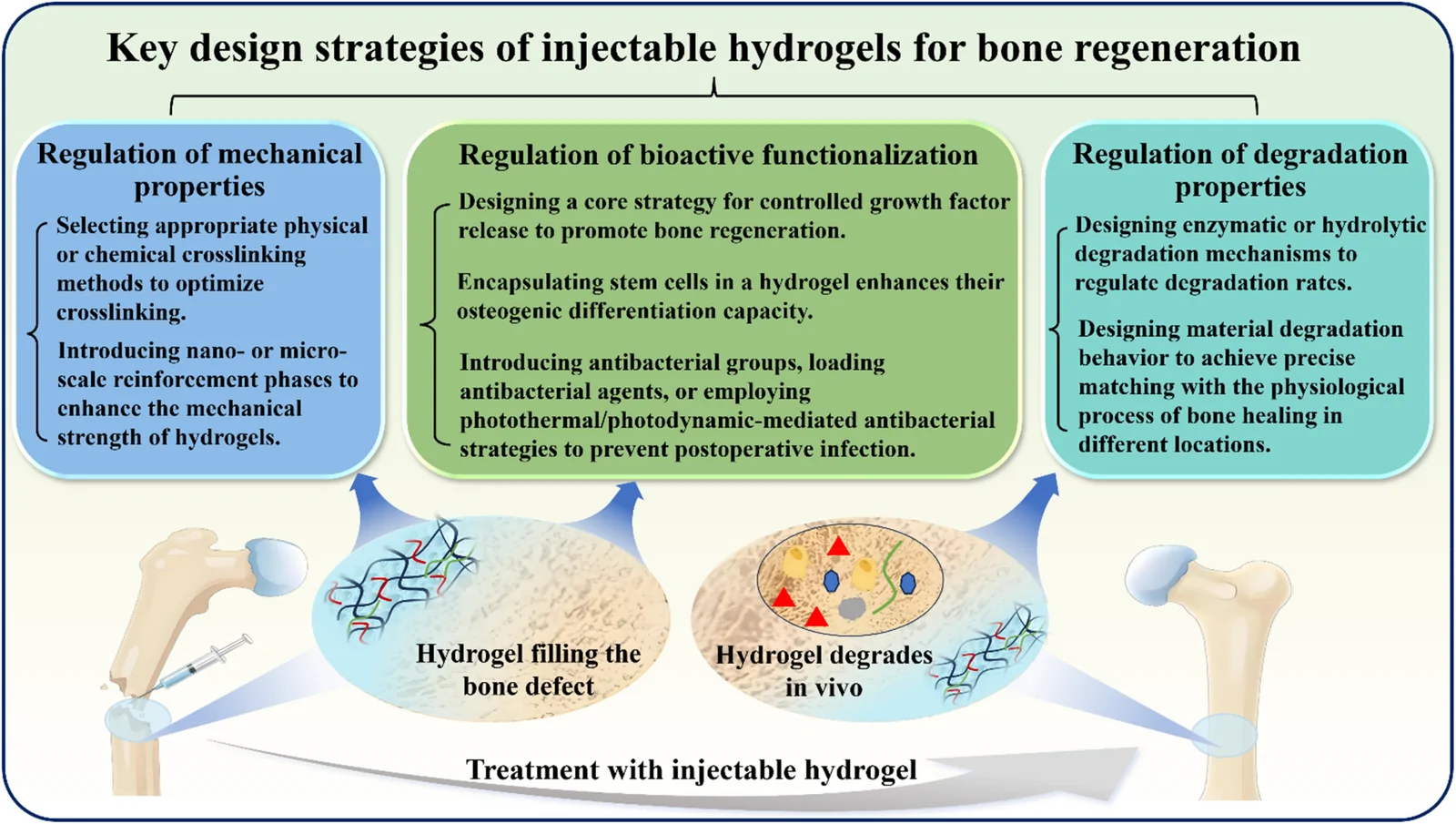
Design strategies for injectable hydrogels for bone regeneration.

The design strategies of injectable hydrogels are based on the entire process of repairing bone defects with hydrogels. The materials need to have flexible and tunable regulation of mechanical properties to adapt to bone defects at different stages, locations, and conditions. To achieve the goal of bone repair, it is necessary to design bioactive functionalized hydrogels that integrate multiple functionalizations, such as controlled release of growth factors and antibacterial properties. Finally, the fully functional hydrogel can degrade safely and timely *in vivo* (Fig. 2).

### 2.1 Manipulation of mechanical properties

Endowing injectable hydrogels with sufficient mechanical properties is a prerequisite for achieving the structural support function in bone regeneration. The mechanical properties required for bone regeneration vary at different stages and locations, necessitating precise regulation of the dynamic adaptation of hydrogels during bone defect repair to ensure that the material can be smoothly injected into the desired site and perfectly fill irregular bone defect cavities.^23–27^ Bone tissue exhibits pivotal anisotropy and gradient characteristics in its mechanical properties.^28^ Song *et al.* reviewed bone regeneration scaffold design strategies and pointed out that when conducting biomimetic design based on the hierarchical structure of natural bone tissue, the significant differences in structural features such as pore size between cortical bone and cancellous bone must be considered, as these differences directly affect mechanical properties.^29^ In actual clinical practice, these crucial mechanical parameters need to be precisely matched with the physiological mechanical ranges of the host's cancellous or cortical bone. If the mechanical properties do not match, stress shielding effects will occur, increasing the risk of early failure such as implant loosening and displacement.^30–32^ Cartilage and intervertebral discs are bone tissues involved in joint movement or spinal motion, which continuously bear complex cyclic mechanical loads such as compression, tension, and shear. Hydrogels need to exhibit stronger fatigue resistance under cyclic mechanical loads.^33^

#### 2.1.1 Optimization of cross-linking methods

The mechanical properties of hydrogels essentially depend on the microstructural characteristics of their cross-linking networks, and different cross-linking methods are the crucial factors influencing this microstructure. Physical cross-linking constructs networks through non-covalent interactions such as hydrogen bonds, hydrophobic interactions, or metal coordination, offering advantages like mild conditions. Combining the characteristics of physical cross-linking with the application requirements of bone repair hydrogels, researchers have proposed optimization strategies for ionic crosslinked hydrogels, hydrogen-bond-crosslinked hydrogels, and hydrophobic interaction crosslinked hydrogels to enhance the physicochemical properties of the materials.

**Fig. 3:**
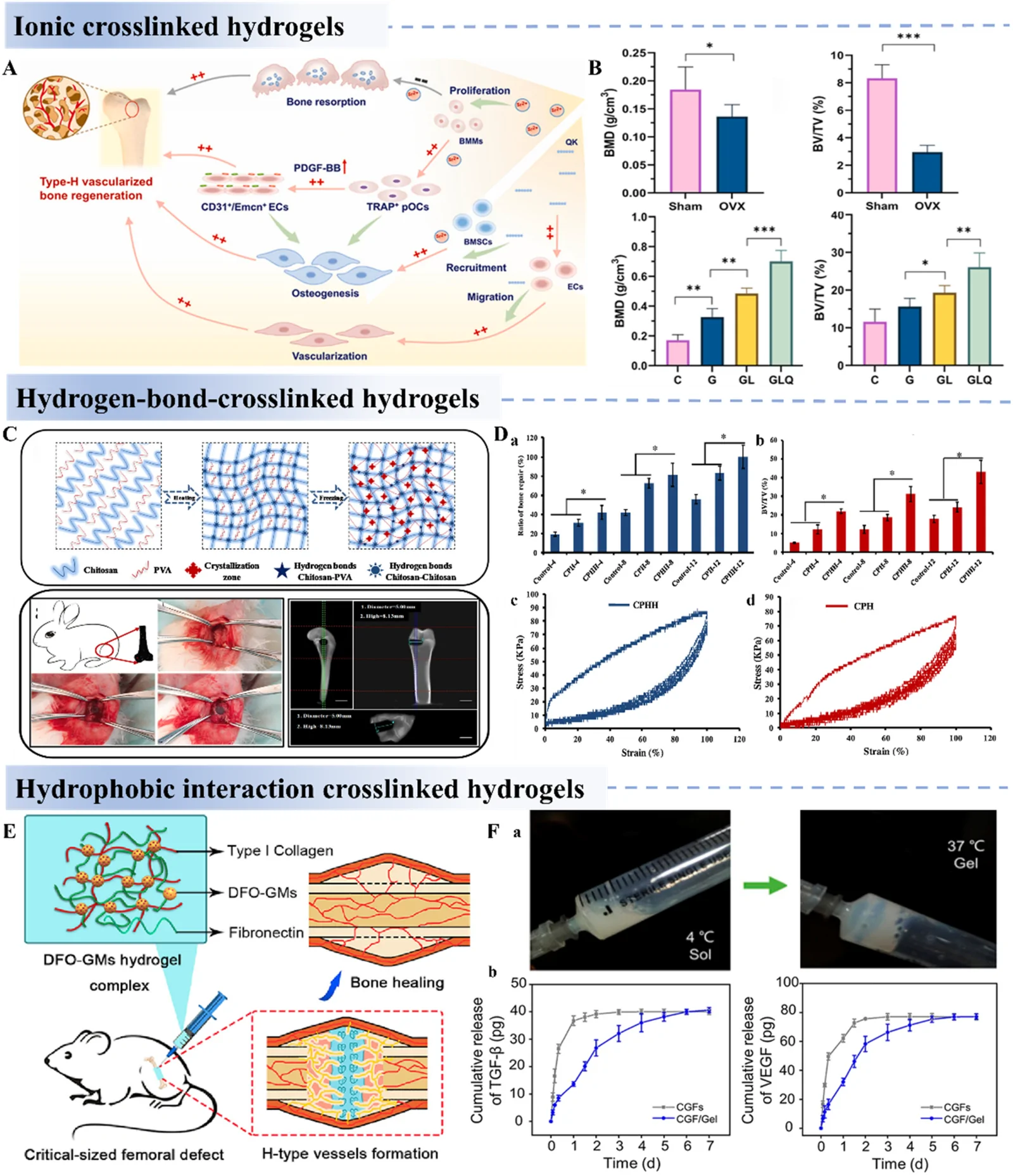
Schematic illustration of physically crosslinked hydrogels and their applications in bone repair, including preparation, mechanisms, and *in vivo* evaluation: (A) schematic illustration of the preparation and mechanism of GelMA-QK/Sr-LDH@PDA multifunctional composite hydrogel. (B) Quantitative results of BMD and BV/TV after *in vivo* osteoporosis modeling and in defect areas. Groups: G (pure GelMA), GL (GelMA/Sr-LDH@PDA), GLQ (GelMA-QK/Sr-LDH@PDA), and C (control). (C) Preparation of CPH; schematic of gel implantation in femoral condyle defects (diameter 5 mm, depth 8 mm) and CT images of defect models (Front, Longitudinal, and Cross sections). (D) (a) Bone repair ratio; (b) BV/TV (*p < 0.05 *vs.* control); (c and d) cyclic tensile tests at 100% strain. (E) Mechanism of action of DFO-GMs hydrogel. (F) (a) Gross images of sol–gel transition; (b) Release profiles of TGF-β and VEGF in CGFs and CGFs-loaded hydrogel (*n* = 3). (A and B) Reproduced with permission. Copyright 2025, Elsevier Ltd.^40^ (C and D) Reproduced with permission. Copyright 2019, Elsevier Ltd.^44^ (E) Reproduced with permission. Copyright 2022, Elsevier Ltd.^48^ (F) Reproduced with permission. Copyright 2022, Frontiers Media S.A.^59^

Ionic cross-linking refers to the formation of reversible cross-linking points between polymer molecular chains through electrostatic interactions of ions with opposite charges, a process that transforms linear polymers from a soluble state into a 3D network gel or solid. This method enables polymers such as sodium alginate to rapidly construct 3D porous hydrogel scaffolds, which can adapt to bone defect areas of different morphologies and exhibit excellent mechanical properties.^34,35^ Leveraging the reversible nature of ionic cross-linking, responsive release of active components in specific microenvironments can be achieved.^36,37^ Researchers have proposed a reverse micelle-mediated strategy, where calcium ions are encapsulated in reverse micelles and delivered to microdroplets, enabling rapid ionic cross-linking for the preparation of sodium alginate hydrogel microspheres.^38^ The method features simple operation and excellent biocompatibility, without the need for acidic conditions or chelating agents, providing a new pathway for the efficient preparation of sodium alginate hydrogel scaffolds. You *et al.* reported a method for preparing polyethylene glycol-alendronate-magnesium (PAMg) hydrogels based on amide formation and metal coordination by mixing alendronate (ALN)-coupled 8-arm polyethylene glycol succinimidyl ester (8-arm-PEG-NHS), magnesium ions (Mg^2+^), and 8-arm polyethylene glycol amine (8-arm-PEG-NH_2_). The ionic cross-linking network adopted by this hydrogel provides robust mechanical support, which can maintain the structural integrity of the gel at the bone defect site, avoid the collapse of the repair material affecting new bone growth, and effectively promote the repair of osteoporotic bone defects.^39^ He *et al.* developed a multifunctional composite hydrogel by incorporating QK peptide and polydopamine-coated strontium-doped layered double hydroxide (Sr-LDH@PDA) into a gelatin methacryloyl (GelMA) matrix, termed gelatin methacryloyl-QK peptide/strontium-doped layered double hydroxide@polydopamine (GelMA-QK/Sr-LDH@PDA), which, through the integration effect related to strontium ion cross-linking, enables strontium-doped layered double hydroxide (Sr-LDH) to exert osteogenic and anti-bone resorption properties (Fig. 3A).^40^ This hydrogel can stimulate the proliferation of bone marrow-derived macrophages, inhibit the maturation and differentiation of osteoclasts, and promote the development of H-type vessels in the bone defect area, significantly promoting bone regeneration under osteoporotic conditions (Fig. 3B).

Hydrogen bond cross-linking forms reversible cross-linking points through hydrogen bond interactions between polymer molecular chains, thereby constructing a 3D network structure. Since hydrogen bond cross-linking simply forms gels through natural intermolecular cross-linking, the resulting materials not only have excellent mildness and biocompatibility but also exhibit high sensitivity to external environmental stimuli during bone repair, enabling “smart regulation”.^41^ Additionally, the dynamic reversibility of hydrogen-bond-crosslinked hydrogels endows them with superior self-healing ability.^42^ While individual hydrogen bonds possess significantly lower bond strength compared to covalent bonds, synergistic effects can substantially enhance the overall performance. For example, Li *et al.* developed a physically double-cross-linked high-strength hydrogel, denoted as P(Am-*co*-Ac-*co*-VDT)/Fe^3+^, by copolymerizing acrylamide (Am), acrylic acid (Ac), and 2-vinyl-4,6-diamino-1,3,5-triazine (VDT) coordinated with ferric ions. This system leverages the synergistic effect of hydrogen bonds and ionic coordination; specifically, the incorporation of VDT enables the formation of hydrogen bonds, while the Ac monomer facilitates carboxylate-Fe^3+^ coordination.^43^ The P(Am-*co*-Ac-*co*-VDT)/Fe^3+^ hydrogel prepared by this method simultaneously possesses robust stability and superior mechanical properties. Bi *et al.* designed a chitosan-polyvinyl alcohol (CPH) physical double-network hydrogel with hydrogen bond cross-linking and crystalline cross-linking by physically cross-linking medical-grade polyvinyl alcohol (PVA) and chitosan in a KOH/urea dissolution system through a simple freeze-thaw process, and then prepared a novel HAp-coated CPH hydrogel (CPHH), in which hydroxyapatite (HAp) nanocrystals accumulated on the surface of the hydrogel through *in situ* mineralization.^44^ The research results showed that this hydrogel exhibited excellent characteristics such as high strength, favorable biocompatibility, cell adhesion, and bone repair capabilities (Fig. 3C and D).

Hydrophobic interaction-induced cross-linking is reflected in the self-assembly behavior of amphiphilic polymers in aqueous environments. Such polymer backbones are simultaneously grafted with hydrophilic and hydrophobic segments. In water, to reduce the system's free energy, hydrophobic segments spontaneously aggregate to form hydrophobic microdomains, which act as physical “cross-linking points” to tether polymer chains into a 3D network. This mechanism endows the material with excellent shear-thinning properties and self-healing ability. Thermosensitive hydrogels widely used in the field of bone repair are essentially the dynamic expression of this hydrophilic-hydrophobic balance changing with temperature, enabling rapid sol–gel transition at the target site.^45,46^ Consequently, the material exhibits excellent injectability, allowing for effective bone defect repair after loading with bone cells or stem cells. Specifically, a previous study utilized additives to induce temperature-responsive sol–gel changes in a multi-component polysaccharide solution, successfully constructing an injectable thermosensitive hydrogel.^47^ In another instance, researchers developed an injectable hydrogel composite system incorporating deferoxamine-loaded gelatin microspheres (DFO-GMs) based on thermosensitive properties (Fig. 3E).^48^ The resulting system capitalizes on these phase transition advantages, undergoing *in situ* solidification at body temperature following injection to perfectly and adaptively fill complex 3D defect morphologies. Combined with the controlled release capability of gelatin microspheres, the extracellular matrix (ECM)-mimicking scaffold not only achieves long-term delivery of deferoxamine but also effectively promotes stem cell infiltration and colonization, thereby constructing a regenerative microenvironment conducive to H-type vessel formation. Zhang *et al.* prepared a local sustained-release system with injectable properties by loading concentrated growth factors (CGFs) into a thermosensitive hydrogel crosslinked with Poloxamer 407.^49^ Poloxamer 407 is a triblock copolymer composed of polyethylene oxide-polypropylene oxide-polyethylene oxide (PEO-PPO-PEO), a typical thermosensitive hydrogel formed through physical cross-linking. CGF-loaded hydrogels containing multiple growth factors can significantly promote the proliferation and osteogenic differentiation of bone marrow mesenchymal stem cells (BMSCs). This hydrogel can undergo a reversible sol–gel transition between 4 °C and 37 °C. After embedding CGFs in the Poloxamer 407 hydrogel, stable and sustained release of transforming growth factor-β (TGF-β) and vascular endothelial growth factor (VEGF) was achieved, effectively promoting BMSCs proliferation and inducing osteogenic differentiation (Fig. 3F).

Physical cross-linking networks possess relatively dynamically reversible properties. When subjected to external stimuli such as temperature changes, pH variations, or light exposure, the cross-linking points undergo breakage and recombination, endowing hydrogels with excellent self-healing ability and shape memory effect, as well as high biocompatibility. This reversibility also means that the cross-linking network is prone to failure when exposed to heat, force, or solvents. Ionic cross-linking, for example, exhibits relatively stable mechanical properties compared to other physical cross-linking methods, but still struggles to meet the high mechanical demands of load-bearing bone defects. In contrast, the covalent bond networks formed by chemical cross-linking often exhibit high stability and rigidity. The internal crosslinks tightly connect polymer chains, enabling the hydrogel to effectively resist deformation and quickly recover its original shape under external force. This characteristic gives hydrogels high gelation speed and high mechanical strength. Among chemical cross-linking methods, photo-cross-linking, Schiff base cross-linking, and click chemistry are the most typical fabrication strategies.

**Fig. 4:**
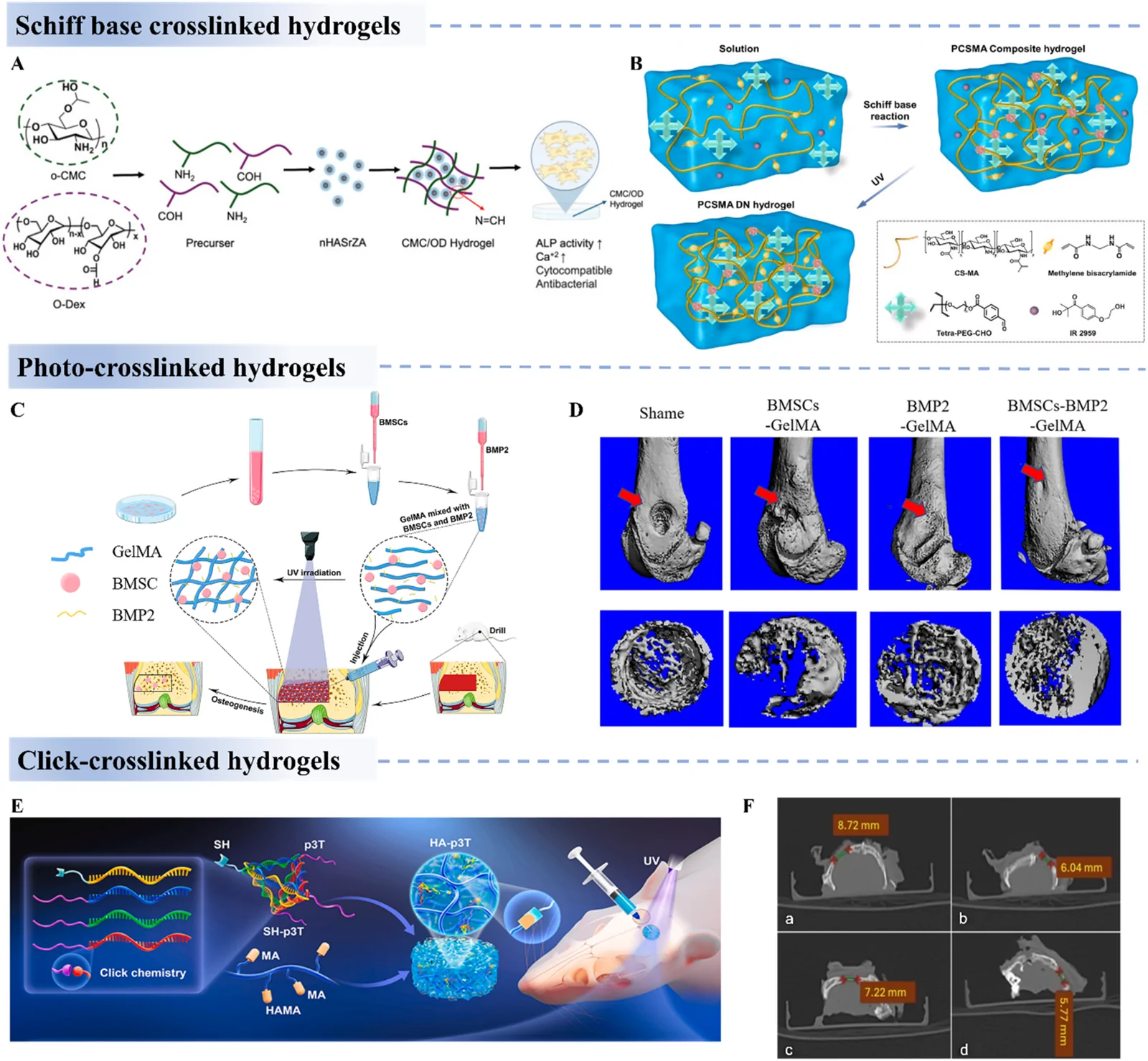
Schematic illustration of chemically crosslinked hydrogels and their applications in bone repair, including preparation, mechanisms, and *in vivo* evaluation: (A) schematic representation of the methodology for the development of composite hydrogels containing zoledronic acid modified strontium hydroxyapatite nanoparticles. (B) Schematic illustration of the fabrication of PCSMA DN hydrogel. (C) Schematic diagram of the preparation of bioactive BMSCs-BMP-2-GelMA scaffold and the experimental procedure. (D) Reconstruction of 3D micro-CT images. (E) Schematic illustration of the construction and mechanism of composite hydrogels (HA-p3T). (F) (a and b) Rabbit 1: diameter of the untreated right bone defect (a) and the left bone defect treated with ELRs (b). (c and d) Rabbit 3: diameter of the untreated right bone defect (c) and the left bone defect treated with ELRs (d). (A) Reproduced with permission. Copyright 2025, Royal Society of Chemistry.^51^ (B) Reproduced with permission. Copyright 2024, Elsevier Ltd.^52^ (C and D) Reproduced with permission. Copyright 2022, Frontiers Media S.A.^55^ (E) Reproduced with permission. Copyright 2025, Elsevier Ltd.^62^ (F) Reproduced with permission. Copyright 2024, Springer Nature.^63^

Schiff base cross-linking is one of the most important cross-linking methods in chemical cross-linking. The principle of this cross-linking method is that the Schiff base reaction can generate unstable imine intermediates, and after other reactions such as hydrolysis and reduction, stable covalent bonds are formed, thus achieving cross-linking between chitosan molecules. Because the Schiff base reaction can often form hydrogels with high biocompatibility, it has been adopted by many researchers and is widely applied in medical and other fields.^50^ Ozgen *et al.* used oxidized dextran and carboxymethyl chitosan as raw materials for Schiff base cross-linking, and incorporated zoledronic acid-modified strontium hydroxyapatite nanoparticles to prepare an injectable hydrogel (Fig. 4A).^51^ This hydrogel not merely exhibits potent antibacterial activity against both *Escherichia coli* and *Staphylococcus aureus* but also promotes osteoblast proliferation, alkaline phosphatase activity, and calcium deposition, thereby facilitating bone regeneration. To optimize the mechanical properties of the hydrogel, relevant studies developed a poly(chitosan methacrylate) double-network (PCSMA DN) hydrogel, which is based on methacrylate-functionalized chitosan derivative and aldehyde-terminated four-arm polyethylene glycol polymer, and is prepared through dynamic covalent Schiff base reaction followed by UV polymerization (Fig. 4B).^52^ Research found that the dynamic balance of Schiff base in the network endows the hydrogel with rapid gelation, easy injectability, excellent tissue adhesion, and pH responsiveness; while the post-construction of the methacrylate cross-linking network significantly improves network density and mechanical properties. Compared with the single-crosslinked poly(chitosan) hydrogel, this double-network structure enhances structural stability, delays degradation rate, and improves mechanical modulus, thereby effectively supporting cell activity, growth, and proliferation, and is beneficial for chondrogenic differentiation.

Photo-cross-linking refers to the cross-linking reaction between polymer chains initiated by active free radicals or cations generated after photosensitizers absorb light energy under ultraviolet and visible light irradiation. In the field of bone repair, photo-cross-linking technology enables the rapid gelation of low-viscosity sol injected into bone defect sites and the rapid construction of covalent cross-linking networks. Such capability not just ensures the effective delivery of drugs and cells but allows precise regulation of its mechanical properties by adjusting conditions such as irradiation time, light intensity, and crosslinker concentration.^53,54^ Building upon these advantages, Chai *et al.* successfully constructed a composite bioactive scaffold of gelatin methacryloyl (GelMA)/BMSCs/bone morphogenetic protein-2 (BMP-2) *via* photo-cross-linking (Fig. 4C).^55^ Through this fabrication strategy, cells and growth factors were efficiently encapsulated under mild conditions, while the light-triggered cross-linking endowed the scaffold with the mechanical strength required for stem cell adhesion and proliferation. Research found that the mechanical microenvironment formed by photo-cross-linking and the biological induction effect of BMP-2 produced a synergistic effect, significantly promoting the osteogenic differentiation of BMSCs (Fig. 4D). *In vivo* imaging and histological studies further confirmed that the scaffold exhibited superior osteogenic potential *in vivo* compared to single-component scaffolds. Another study used poly(l-glutamic acid) (PLGA) with side-chain grafted poly(ethylene glycol) acrylate (APEG) (PLGA-*g*-APEG) as a precursor to prepare hydrogels by ultraviolet light cross-linking, and investigated the effects of APEG grafting rate and PLGA-*g*-APEG precursor mass concentration on the mechanical properties of photo-crosslinked hydrogels.^56^ When the APEG grafting rate was 44.7% and the mass fraction of PLGA-*g*-APEG was 15%, the hydrogel exhibited optimal mechanical properties.

Click chemistry, as an important method in modern organic synthesis, was first proposed and developed by the team of Professor K. Barry Sharpless, a Nobel laureate in chemistry.^57^ The prominent feature of this synthetic strategy is the use of highly efficient and specific chemical reactions to precisely couple molecular structures, thereby constructing functional molecules with complex structures.^58^ This reaction system typically exhibits mild reaction conditions, excellent product yields, and superior biocompatibility, making it of great value in interdisciplinary fields such as biomedical engineering and functional materials science.^59^ In the construction of hydrogels for bone repair, click chemistry technology mainly forms stable 3D network structures through selective intermolecular cross-linking reactions.^60^ Taking the classic copper-catalyzed azide–alkyne cycloaddition (CuAAC) reaction as an example, under the mediation of copper ions, the azide group (–N_3_) and alkyne group (–C≡C) undergo a cyclization reaction to generate the thermodynamic stability of the 1,2,3-triazole ring structure.^61^ Such *in situ*-formed cross-linking network not merely has ideal mechanical properties but also can serve as a controlled-release carrier for bioactive molecules, effectively promoting bone tissue regeneration. Beyond CuAAC, researchers frequently employ derivative reaction systems such as strain-promoted azide–alkyne cycloaddition (SPAAC), inverse electron-demand Diels–Alder reactions, and photo-induced cycloadditions. Leveraging the efficiency and specificity of azide–alkyne click chemistry, a relevant study successfully anchored bone-binding peptides (BBPs) onto tetrahedral framework nucleic acid (tFNA) surfaces to construct BBPs-modified tFNA (BBPs-tFNA) complexes.^62^ Such functionalization not merely achieves the ordered arrangement of BBPs in spatial tissues but also significantly improves the binding efficiency to BMP-2. Subsequently, these BBPs-tFNA complexes were integrated into a methacrylated hyaluronic acid hydrogel system to form a functional bioactive composite scaffold (Fig. 4E). This scaffold can precisely capture endogenous BMP-2, thereby effectively stimulating osteogenic differentiation and facilitating *in situ* bone regeneration. Ruggieri *et al.* innovatively developed a metal-free catalytic click gel system based on elastin-like recombinant polymers (ELRs).^63^ In this platform, the spontaneous cross-linking of functionalized ELRs with reactive polymers under physiological conditions generates biomimetic three-dimensional 3D scaffolds. Experimental validations confirmed that the resulting hydrogel significantly promotes the proliferation and osteogenic differentiation of bone marrow stromal stem cells. The results of animal experiments are more direct evidence. In a rabbit cranial defect model, after 90 days of implantation, micro-CT and histological evaluations confirmed that the newly formed bone tissue in the hydrogel group was not only considerable in quantity but also well integrated with the host bone (Fig. 4F), fully demonstrating its application prospects in bone regenerative medicine.

Chemical cross-linking relies on high-bond-energy covalent bonds to form a 3D network structure. Once the cross-linking is formed, it is irreversible, exhibiting excellent mechanical properties, heat resistance, and solvent resistance, which also implies that it cannot be reprocessed or repaired. During the chemical cross-linking process, cross-linking agents serve as pivotal substances that initiate or promote the formation of covalent bonds between polymer chains. After the cross-linking reaction is completed, if the residual cross-linking agents and their derivatives are not fully removed, they may pose potential hazards to human health.

#### 2.1.2 Integration of reinforcement phase

Introducing foreign reinforcement phases can effectively improve the mechanical strength and biological functions of materials. According to the size characteristics of the reinforcement phase, it can be divided into two major categories: nanoscale and microscale. Nanoreinforcement materials have high specific surface area and excellent mechanical properties. This prominent feature enables them to form intimate interfacial bonding with the hydrogel matrix, thereby enhancing the overall mechanical properties of the hydrogel.^64–66^ Among them, three types of nanomaterials, namely nano-hydroxyapatite (nHA), carbon nanotubes (CNTs), and graphene oxide (GO), have received extensive research and application due to their unique physicochemical properties and biological functions.

**Fig. 5:**
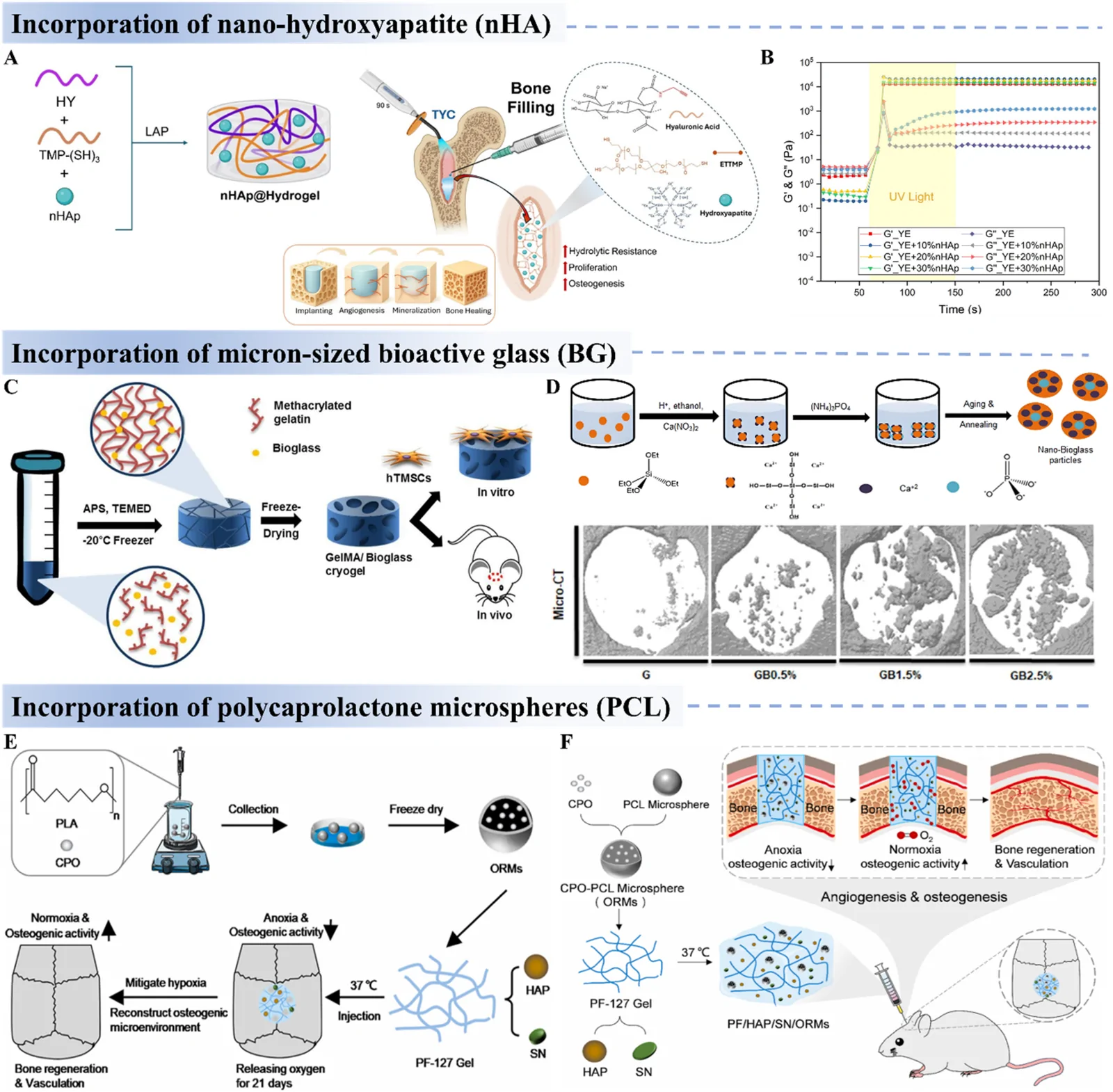
Schematic illustration of hydrogels incorporating different reinforcement phases for bone repair, including preparation and mechanisms: (A) schematic of TYC-based HY hydrogel preparation and mechanism. (B) *In situ* photo-rheological behavior of YE hydrogels containing different nHAp fractions. (C) Experimental scheme for nanobioglass-embedded GelMA cryogels. (D) Nanobioglass synthesis schematic and micro-CT of a 4 mm cranial defect. (E) Schematic of the thermosensitive hydrogel combining CPO-loaded PCL microspheres and hydroxyapatite/LAPONITE® nanoparticles. (F) Schematic of the composite hydrogel for O_2_ release and bone activity promotion. (A and B) Reproduced with permission. Copyright 2025, American Chemical Society.^69^ (C and D) Reproduced with permission. Copyright 2018, MDPI AG.^82^ (E and F) Reproduced with permission. Copyright 2024, Elsevier Ltd.^85^

nHA is the main component of inorganic minerals in human bone tissue, and its crystal structure is highly similar to natural bone apatite, so nHA has excellent bioactivity and osteoconductivity; when nHA is introduced into the hydrogel matrix, the hydroxyl groups on its surface can form hydrogen bonds or electrostatic interactions with hydrogel molecular chains, effectively improving the mechanical properties of the composite hydrogel.^67^ The inorganic composition of nHA similar to natural bone provides an ideal biological microenvironment for the adhesion, proliferation, and differentiation of osteoblasts, promoting bone regeneration and healing.^68^ The nanoscale size effect of nHA can significantly increase the specific surface area of the material, promote protein adsorption and growth factor fixation, thereby accelerating the mineralization process in bone defect areas. Due to the high brittleness and powdery nature of nHA, researchers combine nHA with various materials to prepare composite biomaterials that enhance its biological properties. To address the shortcomings of biological hydrogels such as easy swelling and rapid degradation, a novel photo-cross-linking system achieved performance breakthroughs by introducing nHA.^69^ Researchers prepared a series of hydrogels synthesized *via* photo-induced thiol-alkyne click chemistry between alkyne-functionalized hyaluronic acid (HA) and ethoxylated trimethylolpropane tri(3-mercaptopropionate) (ETTMP) (Fig. 5A). This inorganic nanocomponent not simply significantly improved the structural stability of the material as a supporting skeleton (Fig. 5B), maintaining its morphological integrity in ex vivo bone specimens for a long time, but endowed the hydrogel with osteoinductive activity. Chen *et al.* further developed the drug-loading advantage of nHA, using UV light cross-linking to prepare an injectable composite hydrogel by combining nHA with VEGF and GelMA.^70^ The addition of nHA not just improved cell compatibility due to its bone-like properties but assisted in achieving long-term sustained release of VEGF and calcium ions, making the composite scaffold superior to pure GelMA in mechanical properties and effectively inducing angiogenesis and osteogenic differentiation. In addition, due to the high surface energy of nHA nanoparticles, they are prone to agglomeration in the hydrogel matrix, leading to stress concentration within the material and affecting the mechanical uniformity and biological activity of the composite hydrogel. To solve this problem, researchers usually adopt surface modification techniques such as silanization and polymer grafting to improve the dispersibility of nHA and enhance its interfacial bonding force with the hydrogel matrix. Wang *et al.* used dopamine surface modification to significantly improve the dispersibility of nHA under acidic conditions, thereby enhancing the thermal stability (decomposing only at 323 °C), network structure strength (storage modulus of 2.7 MPa), and internal friction capacity (loss factor of 0.041) of zwitterionic hydrogels.^71^ The benzene ring forms covalent bonds with the hydrogel polymer chains, increasing the compressive strength of the composite hydrogel to 11.66 MPa, which is 32 times that of pure hydrogel.

CNTs as a one-dimensional nanocarbon material, have ultra-high tensile strength and elastic modulus, and their mechanical properties far exceed those of traditional metals and ceramic materials, which can provide excellent mechanical enhancement effects for the hydrogel matrix.^72^ After introducing CNTs into the hydrogel, their unique tubular structure can form a 3D network skeleton inside the material, effectively transferring external stress and significantly improving the compressive and tensile properties of the composite hydrogel, enabling it to better match the mechanical requirements of bone tissue. In addition, the tube walls of CNTs can load bioactive molecules such as growth factors and antibiotics through covalent or non-covalent modification to achieve controlled-release delivery of drugs, providing continuous biological stimulation for the bone repair process.^73^ For example, aminated CNTs can efficiently load BMP-2, which is slowly released during hydrogel degradation, promoting the differentiation of osteoblasts and the deposition of bone matrix. However, the hydrophobic surface and potential cytotoxicity of CNTs limit their application in the biomedical field, and surface functionalization modification, such as carboxylation and polyethylene glycolization, is needed to improve their biocompatibility and dispersibility and reduce their adverse effects on cells.^74^

GO, as an oxidized derivative of graphene, has a surface rich in oxygen-containing functional groups such as hydroxyl, carboxyl, and epoxy groups, giving it favorable hydrophilicity and chemical reactivity. It can form various strong interactions with hydrogel molecular chains, including hydrogen bonds, covalent bonds, and electrostatic interactions, thus effectively improving the mechanical properties and stability of composite hydrogels. Studies have shown that the interfacial energy of composites in the presence of –OH, COOH, and –NH_2_ is more than three times that in the presence of C=O and COC, significantly enhancing interfacial bonding.^75^ The two-dimensional sheet structure of GO can construct a physical cross-linking network inside the hydrogel. Through stacking and entanglement between sheets, it significantly enhances the shear resistance and anti-swelling properties of the material.^76^ At the same time, the large specific surface area of GO can efficiently adsorb bioactive molecules such as proteins and growth factors, achieving their fixation and controlled release in the hydrogel, providing multi-dimensional biological regulation for the bone repair process. In addition, GO also has certain antibacterial activity, which can inhibit bacterial growth by damaging bacterial cell membranes and inducing oxidative stress, reducing the risk of infection in bone defect areas.^77^ Research shows that composite hydrogels with GO introduction can effectively inhibit the proliferation of common pathogenic bacteria such as *Staphylococcus aureus* and *Escherichia coli*, providing a clean microenvironment for bone tissue regeneration.^78^ However, the biosafety of GO still needs further evaluation, especially its long-term *in vivo* toxicity and metabolic behavior, which require verification through more *in vivo* experiments.

Micron-sized reinforcement materials are mainly used to construct porous structures, which are conducive to cell infiltration and nutrient exchange. Researchers design the porous architecture of micron-sized reinforcement materials, regulate their degradation characteristics, and enhance cell responses to improve the overall efficacy of bone repair hydrogels. Among them, micron-sized bioactive glass (BG) and polycaprolactone (PCL) microspheres are often used as micron-sized reinforcement materials for bone repair hydrogels.

Micro-BG particles have unique surface activity. After implantation in the body, they undergo rapid ion exchange reactions, releasing ions such as Ca^2+^ and Si^4+^. These ions can promote the proliferation and differentiation of osteoblasts, induce the deposition and mineralization of bone matrix, and achieve the regeneration and repair of bone tissue.^79^ BG particles themselves also possess excellent osteoconductivity, which can guide the growth of host bone tissue along the surface of the material and accelerate the repair process of bone defect areas.^80^ By physically mixing or covalently binding micro-BG particles into bone repair hydrogels, a porous structure matching the natural pore size of bone tissue is constructed, which is conducive to the infiltration and proliferation of osteoblasts.^81^ Concurrently, the introduction of BG particles can also improve the mechanical properties of hydrogels, especially compressive strength and elastic modulus, enabling them to better withstand the mechanical load during the bone repair process. The research team of Kwon, S. constructed a GelMA cryogel loaded with nano-BG.^82^ This system successfully integrated the ECM simulation characteristics of cryogels with the mineralization-inducing function BG. Through synergistic effects, it significantly enhanced the osteogenic differentiation ability of human tonsil-derived stem cells and confirmed its effectiveness in promoting bone regeneration in a mouse cranial defect model (Fig. 5C and D).

PCL is a synthetic polymer material with favorable biocompatibility and degradability, and its degradation cycle is approximately two to four years, which basically matches the repair cycle of bone tissue.^83^ Micron-sized PCL microspheres have a regular spherical structure and controllable particle size distribution, which can be prepared by methods such as emulsion solvent evaporation and spray drying. Introducing PCL microspheres into bone repair hydrogels and combining them with bioactive ceramics (hydroxyapatite) and natural polymers (collagen, chitosan, gelatin) can significantly enhance the mechanical properties of hydrogels. The spherical structure of PCL microspheres can be evenly dispersed in the hydrogel matrix, effectively transferring mechanical load and reducing stress concentration, thereby improving the compressive strength and elastic modulus of the hydrogel.^84^ In addition, PCL microspheres can also be used as drug-controlled release carriers to load bioactive molecules such as growth factors and antibiotics, achieving slow release of drugs and providing continuous biological stimulation for the bone repair process. Work conducted by Xu *et al.* embedded calcium peroxide (CPO) in PCL microspheres and introduced them together with hydroxyapatite/LAPONITE® nanoparticles into a thermosensitive hydrogel system (Fig. 5E).^85^ This design utilized the embedding characteristics of PCL microspheres to achieve stable oxygen release for up to 21 days, ensuring the survival and migration of stem cells in a hypoxic environment. At the same time, it synergized with the osteoinductive properties of nanoparticles to successfully achieve vascularization and bone regeneration in a rat cranial defect model (Fig. 5F), fully demonstrating the application potential of PCL microspheres in the construction of multifunctional bone repair carriers.

### 2.2 Bioactive functionalization

The repair and regeneration of bone tissue heavily depend on the dynamic balance of the local microenvironment.^86^ This microenvironment system is composed of multiple key elements working synergistically, including heterogeneous cell populations with regulatory functions, structurally complex ECM, and bioactive factors. Osteoblasts and osteoclasts maintain bone metabolism balance through coupling effects, while mesenchymal stem cells (MSCs) differentiate into the osteogenic lineage under the guidance of different signals; in addition to bearing mechanical support functions, the dynamically changing biochemical components of the ECM can also mediate cell responses *via* integrins. To endow hydrogels with biological activity, it is necessary to design the responsive release of growth factors loaded in them within the microenvironment, regulate stem cell colonization, and integrate antibacterial functions.

#### 2.2.1 Selection and release control strategies of core factors

Growth factors play a crucial role in the microenvironment, and their rational controlled release determines the coordinated progression of pivotal processes in bone regeneration, such as angiogenesis, osteoblast differentiation, and the transition from cartilage to bone.^87^ Different factors usually have distinct functions and are suitable for different stages of bone regeneration, so it is necessary to screen for targeted functional factor combinations to achieve precise matching. On the other hand, excessive release of growth factors leads to significant loss of biological activity and other side effects, while insufficient release makes it difficult to achieve the required rate and effect of bone repair; thus, regulating the “on-demand release” of growth factors is equally important.^88^ Among numerous growth factors, three representative core functional factors are selected for detailed analysis. VEGF is responsible for inducing neovascularization and supplying oxygen and nutrients for repair; BMP-2/BMP-7 are the subtypes with the strongest osteogenic activity, capable of directly initiating bone tissue regeneration; TGF-β can not only promote cell proliferation and matrix synthesis but also regulate inflammation and enhance the repair effects of the former two.^89–92^

VEGF induces the proliferation and migration of vascular endothelial cells, thereby promoting the formation of capillaries. It also provides sufficient oxygen and nutrients for newly formed bone tissue, a characteristic that effectively addresses the repair failure caused by insufficient blood supply in large-sized bone defects.^93^ To achieve the synergistic effect of angiogenesis and bone regeneration, precise regulation of VEGF release time is needed to match the speed of bone regeneration. Bone morphogenetic protein (BMP-2/BMP-7) is the core factor for inducing osteogenic differentiation; it activates the Smad signaling pathway within cells, guiding MSCs to differentiate toward osteoblasts.^94^ This function effectively improves the formation efficiency of trabecular bone and the degree of bone tissue mineralization, playing a huge role in the process of bone defect repair. Bone morphogenetic proteins include two functional proteins, BMP-2 and BMP-7, each with different advantages. In the early stage of bone repair, BMP-2 exhibits stronger osteogenic induction activity, while BMP-7 mainly acts on the later repair and remodeling stages of bone tissue. If these two proteins are used in combination, they can act synergistically throughout the entire osteogenic differentiation cycle. TGF-β is more suitable for regulating the process of cartilage-to-bone transition. It promotes the proliferation of chondrocytes and the synthesis of ECM, then accelerates the progression of cartilage-to-bone development. Meanwhile, it can regulate the arrangement and deposition of collagen fibers, enhancing the mechanical stability of newly formed bone tissue.^95^ The coordinated action of these two characteristics allows TGF-β to play an important role in the cartilage formation stage of bone repair. In existing research, three strategies—microsphere encapsulation, heparin affinity binding, and smart responsive release—are widely applied to the controlled release process of growth factors in the microenvironment.

**Fig. 6:**
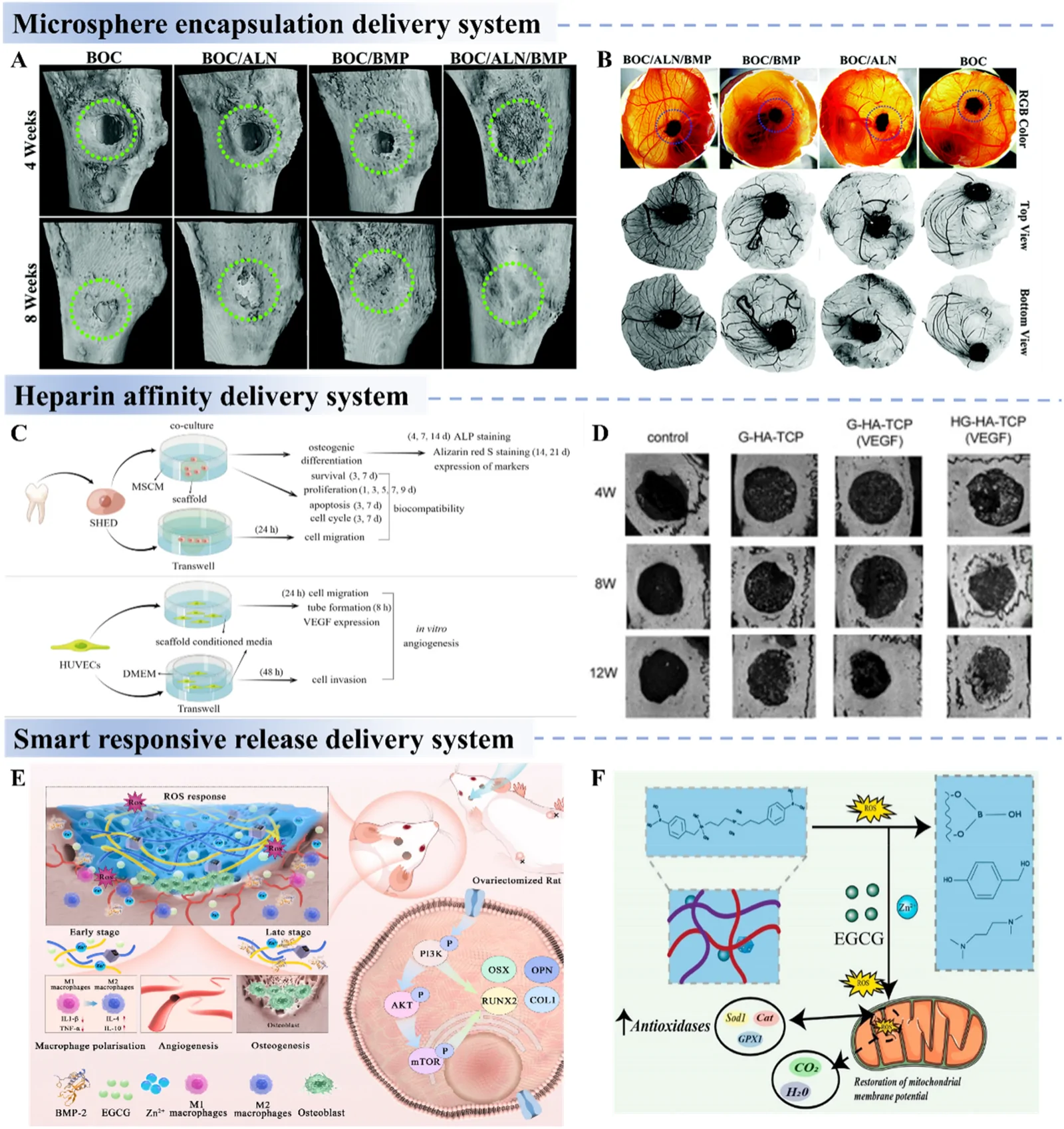
Schematic illustration of various delivery systems for bone repair, including microsphere encapsulation, heparin affinity, and smart responsive release: (A) representative 3D reconstruction of tibial bone defects analyzed by micro-CT. (B) Angiogenesis by CAM assay at three days. (C) Schematic of VEGF-loaded heparinized gelatin-HA-TCP scaffold. (D) Micro-CT observation of bone regeneration. (E) E + B@TZ + Gel preparation for osteoporotic defect repair *via* anti-inflammatory, angiogenic, and osteogenic effects. (F) Mechanism of E + B@TZ + Gel regulating ROS in MC3T3-E1 cells. (A and B) Reproduced with permission. Copyright 2021, Royal Society of Chemistry. B.^97^ (C and D) Reproduced with permission. Copyright 2022, Frontiers Media S.A.^98^ (E and F) Reproduced with permission. Copyright 2025, Elsevier Ltd.^100^

Microsphere encapsulation involves using biodegradable polymer materials such as PLGA and chitosan as microsphere carriers, encapsulating the growth factors to be loaded inside the microspheres or in their porous structures. The gradient degradation characteristics of the microsphere matrix enable the sustained release of growth factors, which is the construction strategy of microsphere encapsulation. This method effectively isolates growth factors from external environmental damage, prolonging the retention time of their biological activity, making it particularly suitable for osteogenic factors like BMPs and VEGF that require long-term effects.^96^ The study by Datta *et al.* addressed the defects of bone ECM hydrogels and developed a microsphere-encapsulated bone-inductive decellularized bone ECM/oleoyl chitosan-based hydrogel construct (BOC).^97^ Through the synergistic effect of gelatin microspheres and the hydrogel matrix, the dual sustained release of growth factor BMP-2 and anti-resorption drug ALN was successfully achieved. This microsphere embedding strategy effectively solved the problems of matrix instability and growth factor loss, significantly promoting stem cell osteogenic differentiation and vascularization (Fig. 6A and B).

Heparin and growth factors possess strong specific affinity, allowing them to be readily incorporated into the hydrogel network. By altering conditions such as pH and ionic strength, target molecules can be triggered to release in a strategy known as heparin affinity-based controlled release. Such an approach prolongs the *in vivo* half-life of growth factors while preserving their biological activity, preventing rapid metabolic clearance. Furthermore, encapsulating heparin-modified gelatin nanospheres pre-loaded with growth factors into larger microspheres establishes a multi-level release system, significantly enhancing osteoinductive capacity. By utilizing these properties, the research team of Chen, X. focused on the functional application of heparin and constructed a heparinized gelatin-hydroxyapatite-tricalcium phosphate scaffold loaded with VEGF (Fig. 6C).^98^ By capitalizing on the natural high affinity between heparin and growth factors, heparin acts as an essential “molecular anchor” within the scaffold, enabling the efficient loading and stable, sustained release of VEGF. Through this specific mechanism, the scaffold effectively maintains the biological activity of VEGF, significantly strengthens the coupling of osteogenesis and angiogenesis, and ultimately accelerates bone regeneration (Fig. 6D). Another study used heparin-chitosan layer-by-layer self-assembly technology to modify PLGA microspheres, constructing a dual-loading system.^99^ They employed microsphere encapsulation to successfully avoid the protein release hindrance caused by electrostatic interactions between positively charged proteins and negatively charged sodium alginate, enabling ordered release of BMP-2 and VEGF, while the presence of heparin enhanced the biological efficiency of the proteins. This construction method significantly improved the spatiotemporal coordination of bone regeneration.

The crucial point of smart responsive release is that the release switch of growth factors can be precisely triggered based on high oxidative stress levels, acidic pH in inflammatory sites, or high expression of specific enzymes. This “on-demand release” strategy not merely effectively prolongs the *in vivo* half-life of growth factors but also achieves precise enrichment of therapeutic factors in specific time and space. Xiao *et al.* addressed the treatment bottleneck of complex and difficult-to-regenerate microenvironments in osteoporotic bone defects, developing a microenvironment-responsive hydrogel delivery system (Fig. 6E) that achieved spatiotemporal sequential release of epigallocatechin gallate (EGCG) and BMP-2.^100^ This system utilizes the high oxidative stress characteristics of the pathological microenvironment to trigger the response. By releasing active factors in stages, it not simply uses EGCG to improve the inflammatory microenvironment and resist oxidative stress in the early stage but continuously induces osteogenic differentiation through BMP-2 in the subsequent stage (Fig. 6F).

#### 2.2.2 Stem cell encapsulation and behavioral modulation

Stem cells are the “seed cells” for bone regeneration, and their osteogenic differentiation potential and *in vivo* survival efficiency directly determine the final outcome of bone repair.^101^ Therefore, it is necessary to screen for stem cell types with strong proliferation ability and high osteogenic differentiation activity. However, in the actual clinical translation, transplanted stem cells often suffer from a large loss of function or even death due to ischemia, hypoxia, or oxidative stress. To solve this problem and ensure the functional integrity of high-potential stem cells, the hydrogel microenvironment of stem cells needs to be set up to be similar to the *in vivo* microenvironment, and this biomimetic microenvironment can be regulated to enhance the colonization and differentiation ability of stem cells.^102^ Combining with the clinical needs of bone repair, three typical stem cell types with excellent osteogenic differentiation performance, BMSCs, adipose-derived stem cells (ADSCs), and induced pluripotent stem cells (iPSCs), are selected for this discussion.

BMSCs have significantly higher expression levels of osteogenesis-related genes than other stromal cells, so they have a natural advantage in osteogenic differentiation and can efficiently induce new bone formation in both *in vitro* and *in vivo* models. The GelMA hydrogel loaded with sirtuin 3 (SIRT3) plasmid prepared by Zhu *et al.* can effectively improve the mitochondrial function of aged BMSCs. The results of animal experiments showed that the bone defect repair efficacy in aged rats was over 40% higher than that of the conventional approach, suggesting that aged BMSCs with restored osteogenic activity can effectively promote bone defect repair.^103^

ADSCs have strong proliferation ability and are widely available. Although their differentiation potential is not as strong as that of BMSCs, they are ideal complementary cells for BMSCs. In other words, when co-cultured with BMSCs, the differentiated cells exert a stronger paracrine effect and synergistically improve osteogenic efficiency. Luo *et al.* confirmed through *in vitro* experiments that the alkaline phosphatase (ALP) activity and calcium deposition of the co-culture group of BMSCs and ADSCs were significantly higher than those of the single-cell group. After eight weeks of *in vivo* implantation, the hydroxyapatite/chitosan/poly(l-lactic acid) (HA/CS/PLLA) scaffold, the new bone formation area reached (497.75 ± 7.44) µm^2^, far exceeding that of the single-cell loading group.^104^ Simultaneously, the study of Zhang *et al.* showed that ADSCs are more sensitive to oxidative stress, but their proliferation and anti-apoptotic abilities can be significantly improved through signal pathway regulation, and they have excellent adaptability.^105^

iPSCs have become a promising source of seed cells in the field of bone repair due to their unlimited proliferation ability and multipotent differentiation potential. Compared with adult stem cells such as BMSCs and ADSCs, iPSCs can be directed to differentiate into functionally mature osteoblasts, and can avoid the differences in cell quality caused by donor age and health status. A previous study evaluated the ability of mouse iPSCs to differentiate into bone, cartilage, and fat *in vitro* and their ability to maintain the osteoblast phenotype on scaffolds.^106^ The results showed that iPSCs could differentiate into the mesenchymal lineage, and the derived osteoblast grafts expressed bone-specific matrix, showing the persistence of mineralization and osteoblast phenotype, and there were vascular recruitment and microvascular formation, proving that iPSCs were functionally differentiated into osteoblasts, providing a cell source for orthopedic medical applications and disease mechanism research.

**Fig. 7:**
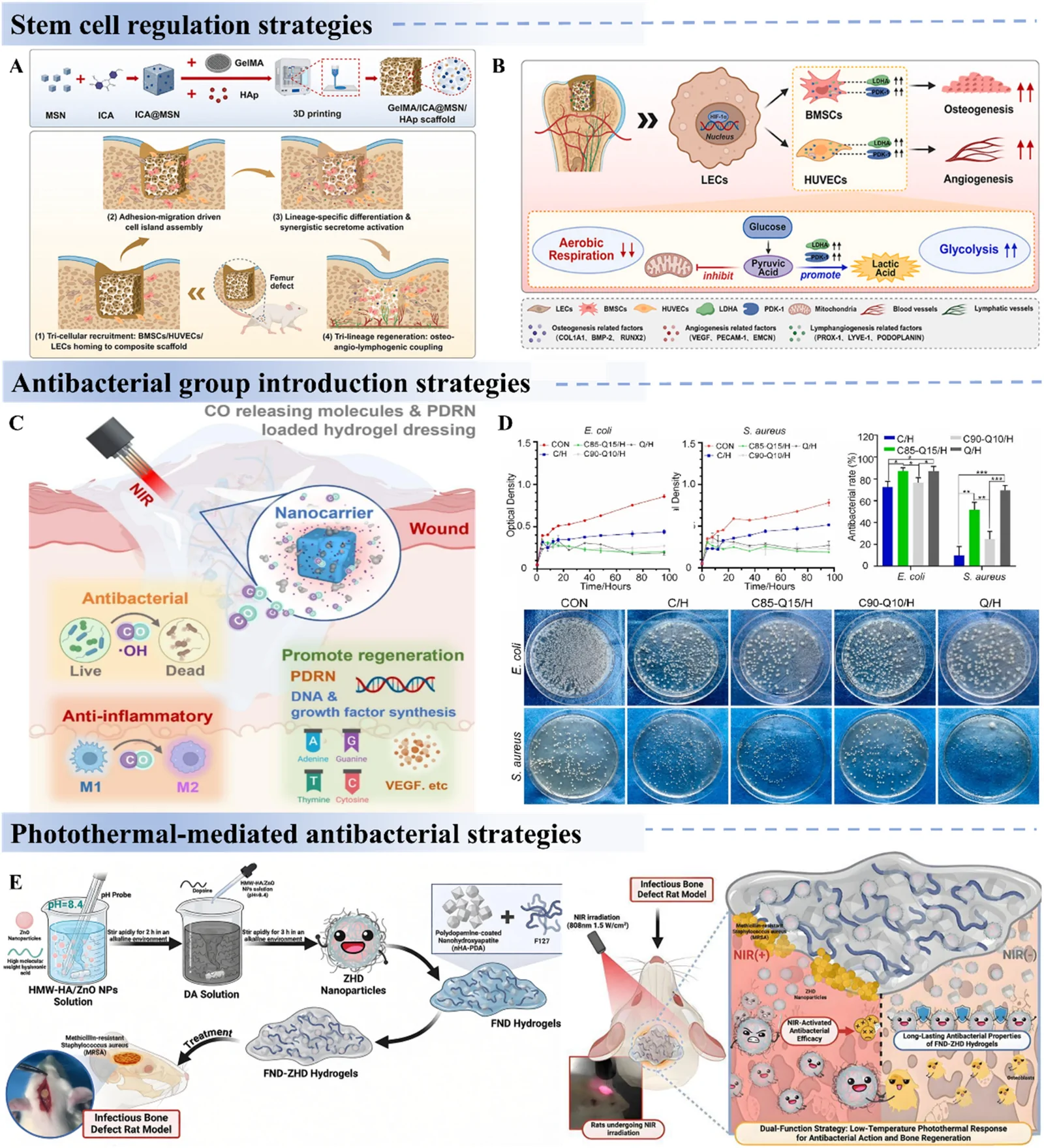
Schematic illustration of stem cell colonization strategies in hydrogel applications: (A) Schematic diagram of the preparation process for the GelMA/ICA@MSN/HAp scaffold and schematic diagram of the *in vivo* action mechanism of the GelMA/ICA@MSN/HAp scaffold. (B) HIF-1α mediated mitochondrial metabolic reprogramming promotes bone repair. (C) Schematic illustration of the mechanism of the NIR-responsive hydrogel for infected wound therapy. (D) Antibacterial activities of C/H, C85-Q15/H, C90-Q10/H, and Q/H against *E. coli* and *S. aureus* after co-culturing for 96 h. Agar plate experiment after 24 h of co-culturing with *E. coli* and *S. aureus*. (E) FND-ZHD prep and mechanism for infectious bone defects. (A and B) Reproduced with permission. Copyright 2026, KeAi.^109^ (C) Reproduced with permission. Copyright 2025, Elsevier B.V.^113^ (D) Reproduced with permission. Copyright 2024, Elsevier Ltd.^114^ (E) Reproduced with permission. Copyright 2025, Wiley.^115^

After stem cell transplantation, the transplanted area needs to rely on the ingrowth of blood vessels from the host tissue to provide nutrients. Before the formation of new blood vessels, the transplanted area will be in an avascular or hypovascular state, and oxygen and nutrients cannot be effectively transported. Or due to local tissue injury caused by transplantation, an inflammatory response is triggered, leading to increased microvascular permeability and slow blood flow, so it is easy to have ischemia and hypoxia. Stem cells may be damaged or even die due to energy depletion. Researchers often adopt the strategy of introducing hypoxia-inducible factors into the gel.^107^ Under hypoxic conditions, HIF-1α can enter the nucleus smoothly, form a heterodimer with HIF-1β in the nucleus, and bind to the hypoxia-response element (HRE) of target genes to achieve the transcription of genes related to angiogenesis (VEGF, Ang-1), energy metabolism reprogramming (GLUT1, LDHA), osteogenic differentiation (RUNX2, COL1A1), and anti-apoptosis (Bcl-2, survivin).^108^ Among them, the formation of new blood vessels improves the blood supply and oxygenation in the defect area, breaking the vicious cycle of hypoxia-apoptosis. The latest research proposed an innovative “bone-vascular-lymphatic triple coupling” strategy and developed a 3D-printed biomimetic scaffold GelMA/ICA@MSN/HAp (Fig. 7A).^109^ They found a new mechanism: the activation of the scaffold can activate lymphatic endothelial cells (LECs), and LECs mediate paracrine signaling under the action of hypoxia-inducible factor 1 (HIF-1α), driving mitochondrial metabolic reprogramming in BMSCs and human umbilical vein endothelial cells (HUVECs). The experimental results showed that the scaffold significantly enhanced the generation of new blood vessels and bone regeneration ability (Fig. 7B). In this process, the HIF-1α pathway is a core element, and LEC-derived C-X-C motif chemokine ligand 12 (CXCL12) is involved in the metabolic reprogramming required for bone formation and vascular differentiation.

During stem cell transplantation, mutations in the cellular microenvironment and inflammatory responses will produce a large amount of reactive oxygen species (ROS), while also leading to mitochondrial dysfunction, damaging the DNA, lipids, and proteins of stem cells. For this oxidative stress situation, vitamin C is a natural water-soluble antioxidant. Its molecular structure contains an enediol group, and the electrons it provides can directly neutralize reactive oxygen species such as superoxide anion and hydroxyl radical, converting them into harmless water molecules and oxygen; concurrently, it can act as an electron donor to promote the regeneration of other antioxidants such as vitamin E and glutathione. Through the process, excess ROS are cleared by vitamin C, solving the problem of oxidative stress and building a stable microenvironment foundation for stem cell survival and function. The study of Zhao *et al.* applied the antioxidant properties of vitamin C to the microenvironment regulation of hypoxic bone defects, and constructed a sodium alginate composite hydrogel loaded with calcium peroxide and vitamin C.^110^ In this system, vitamin C acts as a pH regulator and antioxidant, releases oxygen to alleviate the hypoxic state in the bone defect area, and reduces the side effects of excessive hydrogen peroxide, providing an effective microenvironment stabilization strategy for stem cell transplantation.

#### 2.2.3 Integration of antibacterial function strategies

Postoperative infection is a common clinical problem. Once it occurs, it may lead to surgical failure of bone repair, loosening and detachment of bone graft materials, and even serious complications such as systemic infection. To obtain better clinical effects, researchers integrate antibacterial functional modules with bone repair hydrogel materials. For example, loading antibacterial agents in hydrogels, introducing antibacterial groups, and targeted sterilization by photothermal and photodynamic methods.

The antibacterial agents commonly used by researchers include gentamicin, vancomycin, *etc.* The 3D network structure of hydrogels has the characteristics of porosity and hydrophilicity, which can well load antibacterial agents into the bone repair site. With the sustained-release regulation method of hydrogels mentioned above, antibacterial agents can be slowly and continuously released at the bone defect site, so that a stable effective antibacterial concentration can be maintained in the local microenvironment. Compared with the traditional method of systemic intravenous infusion of antibiotics, it will not cause insufficient drug concentration in the local bone defect area, drug overdose in the systemic circulation leading to hepatic and renal function damage, and bacterial drug resistance caused by long-term abuse. Previous work constructed a pH-responsive GelMA-OSA hydrogel, which physically blended and loaded gentamicin sulfate (GS).^111^ By using the acidic characteristics of the infection site, the precise release of GS was achieved, showing high antibacterial activity against *Staphylococcus aureus*. On the basis of effectively clearing the infection barrier, the system further used mesoporous silica nanoparticles to encapsulate the osteogenic drug phenamil (Phe) to synergistically enhance osteogenic differentiation. *In vivo* experiments confirmed that this dual-functional hydrogel led by “antibacterial agent loading” significantly promoted new bone formation.

Introducing antibacterial groups into hydrogels is a safe and effective antibacterial strategy. Because this antibacterial strategy does not use antibiotics, it avoids the risk of drug resistance from the source. Researchers use chemical modification methods to directly covalently bind functional groups (such as quaternary ammonium groups, betaine groups, *etc.*) with antibacterial activity to the polymer backbone or side chains of hydrogels. When bacteria contact the surface of the hydrogel, these antibacterial groups will interact with the bacterial cell membrane, destroy the structural integrity or metabolic function of bacteria, and achieve the purpose of sterilization. If the structure and modification density of the groups can be precisely regulated, a balance between antibacterial activity and biocompatibility can be achieved. Quaternary ammonium groups serve as a safe, efficient, and multifunctional gene delivery platform, as well as one of the most widely used antimicrobial modification moieties to date. Quaternary ammonium groups carry positive charges, while the surface of bacterial cell membranes is negatively charged.^112^ Through electrostatic interaction, they can quickly adsorb to the surface of bacterial cell membranes. The hydrophobic long chains of quaternary ammonium groups insert into the phospholipid bilayer of the cell membrane, destroying the stability and integrity of the cell membrane, and ultimately killing the bacteria. Based on this mechanism, to further enhance the intervention ability in complex infected microenvironments, our team designed a dual-functional antibacterial hydrogel with “static contact + dynamic release” in a previous study (Fig. 7C).^113^ The study used cation-enhanced carboxymethyl chitosan (CMC) as the gel matrix to provide a basic contact-based bactericidal barrier, and at the same time introduced Prussian blue (PB) nanoparticles loaded with carbon monoxide-releasing molecules (CORMs). Under the trigger of near-infrared (NIR) light, the release of CORMs was achieved. This method synergistically inhibits bacteria through static cationic groups and dynamically released CO gas, and combines with the active factor polydeoxyribonucleotide (PDRN) for immune regulation, effectively disrupting the cycle of infection and inflammation, and achieving high-quality repair of infected wounds. Tian *et al.* designed a dual-functional, thermosensitive, and injectable hydrogel composed of chitosan, quaternized chitosan (QCS), and nHA, and optimized the ratio of CS to QCS in the hydrogel to enhance the antibacterial effect of CS while reducing the cytotoxicity of QCS.^114^*In vitro* studies showed that the hydrogel with a CS to QCS ratio of 85%:15% exhibited excellent biocompatibility and antibacterial performance (Fig. 7D), and also had appropriate mechanical properties and degradability. The incorporation of nHA into the hydrogel can promote the proliferation and osteogenic differentiation of MC3T3-E1 cell. This hydrogel showed superior *in vivo* therapeutic effects in a rabbit infectious bone defect model.

The most prominent feature of photothermal/photodynamic-mediated antibacterial mechanisms is the realization of “on-demand antibacterial”, that is, performing light source irradiation in the required area and time to exert targeted antibacterial effects. This method can effectively avoid damage to surrounding normal tissues and stem cells. Photothermal/photodynamic-mediated antibacterial mechanisms involve integrating light-responsive nanomaterials (such as black phosphorus nanosheets, GO, *etc.*) into the hydrogel system. These nanomaterials usually have photothermal conversion effects or photodynamic effects. Under the irradiation of light sources with specific wavelengths (such as near-infrared light), spatiotemporal precise killing of pathogenic bacteria in bone defect areas can be achieved. The introduced nanomaterials can also endow hydrogels with superior mechanical properties and osteoinductive activity, achieving multifunctional synergy. A recent study reported an injectable nanohybrid hydrogel (FND-ZHD) featuring an efficient and safe photothermal-mediated antibacterial strategy.^115^ By incorporating functional components such as polydopamine, the formulation exhibits robust photothermal conversion under near-infrared irradiation, enabling precise, low-temperature therapy that effectively eradicates resistant pathogens such as MRSA (Fig. 7E). Notably, the treatment synergistically generates reactive oxygen species (ROS), significantly amplifying the overall antibacterial efficacy. Meanwhile, the intelligent design of the hydrogel can clear excess ROS, thus solving the potential biotoxicity problem of photothermal therapy while ensuring strong bactericidal effects. Chen *et al.* prepared a porous collagen (Col) scaffold enhanced by two-dimensional GO and black phosphorus (BP).^116^ The Col-GO@BP scaffold exhibits efficient photothermal antibacterial effects under NIR irradiation, and significantly promotes the osteogenic differentiation of BMSCs by utilizing its mild photothermal effect and biomineralization process.

### 2.3 Modulation of degradation behavior

In the clinical practice of bone defect repair, bone healing sequentially goes through the inflammatory phase, proliferative phase, and remodeling phase, and the adaptability of materials to the dynamic process directly determines the outcome of bone repair. However, traditional bone repair materials (such as metal implants, inert bioceramics) fail to meet this requirement: metal materials have sufficient mechanical strength but cannot degrade, and their long-term retention in the body also triggers inflammation; although bioceramics can degrade, their degradation rate does not match the rhythm of bone regeneration—excessive degradation leads to premature loss of mechanical support, causing problems such as collapse of new bone and fracture displacement; too slow degradation forms a physical barrier, hindering the migration of osteoblasts and vascular invasion, ultimately leading to repair failure. Therefore, to make the degradation behavior of materials adapt to the dynamic physiological process of bone healing sites, precise degradation mechanism design and rate regulation based on the mechanical needs of different bone repair sites are provided for hydrogels here.

#### 2.3.1 Design strategies for controlled degradation

Currently, the main degradation mechanisms include specific enzyme-mediated degradation and non-specific hydrolytic degradation. Enzyme-mediated degradation is a biodegradation method that relies on the catalytic action of specific enzymes in the body to achieve controllable degradation of materials, which can precisely respond to changes in the tissue microenvironment and provide dynamically adaptable scaffold structures for the bone repair process. Hydrolytic degradation refers to the degradation mode in which the molecular chains of materials break under the action of water, and its degradation rate mainly depends on factors such as the chemical structure, molecular weight, and cross-linking degree of the materials. Precisely regulating and designing these two degradation methods is extremely important for achieving a perfect match between degradation and the dynamic physiological process of healing.

Enzyme-mediated degradation mainly relies on the action of *in vivo* enzymes for degradation regulation. Gelatinases such as MMP-2 and MMP-9 begin to be expressed in the inflammatory phase, reach their peak in the repair phase, and then gradually decline. This temporal expression pattern provides endogenous trigger signals for designing intelligent degradable materials.^117^ The most typical method of enzyme-mediated degradation strategy is to introduce matrix metalloproteinase (MMPs)-sensitive peptide bonds. During the bone tissue repair process, the expression of MMPs changes with different healing stages. Researchers can design peptide bonds sensitive to specific MMPs. When these enzymes are expressed at the bone healing site, they can specifically recognize and cleave the peptide bonds in the material, thus achieving enzyme-mediated degradation of the material. This degradation mode can be highly synchronized with the natural repair process of bone tissue. While bone tissue gradually regenerates, it can also achieve the orderly release of bioactive components, providing continuous support for the growth and differentiation of bone cells. Wu *et al.* developed an enzyme-responsive deoxyribonucleic acid (DNA)–polyethylene glycol (PEG) hydrogel.^118^ The hydrogel integrates a PEG network cross-linked by MMP-cleavable peptide bonds and covalently links VEGF-binding DNA strands stabilized by actin, successfully achieving spatiotemporal controllable release of therapeutic drugs. After implantation, MMPs can promote hydrogel degradation, and VEGF is released to promote osteogenic differentiation and angiogenesis. The actin-stabilized DNA framework can maintain structural integrity at this stage and prevent premature release of phosphate. Subsequently, matrix remodeling releases nucleases, which catalyze DNA to generate phosphate ions. These phosphate ions, together with the peptides in the hydrogel and endogenous calcium, synergistically drive mineralization.

Factors affecting the hydrolytic degradation rate mainly include the ester bond density of synthetic polymers and the cross-linking degree of natural polymers. For synthetic polymer materials, such as adjusting the ratio of lactic acid (LA) and glycolic acid (GA) in PLGA, the ester bond density of PLGA can be changed. The higher the ester bond density, the slower the hydrolytic degradation rate of the material; conversely, the faster the degradation rate. For natural polymer materials like hyaluronic acid, the higher the cross-linking degree, the tighter the material structure, and the slower the hydrolytic degradation rate; the lower the cross-linking degree, the relatively loose structure, and the faster the degradation. Based on this premise, as long as these parameters are precisely controlled, the hydrolytic degradation of the material within a specific time can be achieved to meet the needs of different bone repair scenarios. The advantage of hydrolytic degradation is strong controllability and high stability. It can achieve precise regulation of the degradation rate through precise design of the material's chemical structure and preparation process, providing stable mechanical support and a bioactive microenvironment for the bone repair process. Its disadvantage is the lack of biological specificity, which makes it impossible to precisely respond to changes in the tissue microenvironment. Therefore, it usually needs to be combined with biological degradation methods such as enzyme-mediated degradation.

#### 2.3.2 Matching degradation rates for different bone sites

Non-load-bearing bones refer to the bones in the human skeletal system whose main function is not to bear body weight or transmit mechanical loads, but mainly to protect internal organs, maintain morphology, and provide muscle attachment points. This type of bone tissue can achieve preliminary healing in a relatively short time, so the repair materials need a relatively fast degradation rate. For example, the skull is situated in the head, primarily functioning to protect brain tissue and preserve cranial shape, and is classified as a non-load-bearing bone. The skull region exhibits relatively abundant blood supply. During skull healing, efficient blood circulation can provide sufficient nutrients and oxygen for the growth and differentiation of bone cells, and also help in the excretion of metabolic wastes. This characteristic makes the healing and regeneration of bone tissue relatively fast, requiring a faster degradation rate. When designing enzyme-mediated degradation mechanisms, MMP-sensitive peptide bonds can be introduced, which can be easily recognized and cleaved by MMPs in the early stage of bone healing to achieve rapid degradation. For materials using hydrolytic degradation, the proportion of GA can be appropriately increased when PLGA degrades, and hyaluronic acid with lower acetylation degree can be selected to accelerate the hydrolytic degradation rate, so as to match the rapid regeneration of bone tissue.

Load-bearing bones such as the tibia and femur still need to continuously bear body weight and mechanical loads generated by movement while healing bone defects, which greatly increases the difficulty and reduces the speed of bone healing. In addition, load-bearing bones need to recover to a functional state that can normally bear body weight and perform various activities, which puts higher requirements on the quality and strength of healing, making the healing process relatively long. In contrast to non-load-bearing bones, methods such as introducing peptide bonds with low sensitivity to MMPs, appropriately increasing the proportion of LA, and selecting hyaluronic acid with higher acetylation degree can be used to achieve slow degradation of the material. Xia *et al.* synthesized a series of PLLA-trimethylene carbonate (TMC) GA ternary copolymers, in which the monomer feeding ratios of l-lactic acid, TMC, and GA were different. The researchers evaluated the effects of different GA contents in PLLA-TMC-GA ternary copolymers on their chain structure and *in vitro* degradation performance.^119^ The experimental results showed that as the GA content increased, the degradation rate of the ternary copolymer accelerated. The reason is that the GA segments with faster hydrolysis rates not only effectively disrupted the regularity of the LLA segments but also increased the probability of chain scission during degradation.

## 3. Matrix materials of injectable hydrogels

The hydrogel matrix, as the core carrier of bone repair materials, directly determines the outcome of bone defect repair. However, single-component hydrogels often have inherent defects such as insufficient mechanical strength or low biological activity. To overcome these performance issues, functional fillers can usually be added to the hydrogel matrix to optimize its comprehensive performance, such as enhancing mechanical strength, improving osteoconductivity, or enhancing cell compatibility, so as to meet the material performance requirements for bone repair.

Based on these optimization strategies, current bone repair hydrogel materials are mainly divided into four major categories, each with distinct material characteristics (Table 1). Natural polymer-based hydrogels have natural compatibility with human tissues and are suitable as carriers for cell adhesion and proliferation, but they usually have insufficient mechanical properties and are more suitable for bone defect repair in non-load-bearing areas. Synthetic polymer-based hydrogels can precisely regulate molecular structure to synchronize the degradation cycle with the bone regeneration process, yet they have low biological activity and need to be improved by surface modification or loading active factors. Natural–synthetic composite hydrogels are the current research focus, combining the biocompatibility of natural materials and the adjustable mechanical properties of synthetic materials, but their production process is relatively complex. Filler hydrogels significantly enhance mechanical support capacity by adding osteoconductive inorganic components, however, the problem of uneven dispersion of inorganic particles restricts their application in injectable molding.

**Table 1:** Classification of bone repair hydrogel materials

Material type	Typical examples	Advantages	Limitations
Natural polymer-based hydrogels	Alginate, hyaluronic acid, gelatin	Favorable biocompatibility, easy functionalization, mimics ECM^120^	Low mechanical strength, difficult to control degradation^121^
Synthetic polymer-based hydrogels	poly(ε-caprolactone-*co*-lactide)-*b*-poly(ethylene glycol)-*b*-poly(ε-caprolactone-*co*-lactide) (PCLA–PEG–PCLA), polyethylene glycol diacrylate (PEGDA)	Strongly adjustable mechanical properties, controllable degradation^122^	Low biological activity, easy to cause inflammation^123^
Natural–synthetic hybrid hydrogels	Gelatin–PEG, chitosan–PLGA	Balances biocompatibility and mechanical properties^124^	Complex preparation, poor batch stability^125^
Filler hydrogels	Hydrogel/HA, hydrogel/BG	Enhances mechanical strength, endows osteoconductivity^126^	Inorganic particles are prone to agglomeration, affecting injectability^127^

### 3.1 Natural polymer

Natural polymers exhibit excellent biocompatibility, non-toxic degradation byproducts, and abundant active sites, making them ideal candidates for injectable hydrogel research.^129^ These hydrogels contain polysaccharides and proteins, rich in active groups such as hydroxyl, amino, and carboxyl groups.^130^ Various bioactive components can be successfully introduced through simple chemical coupling or physical adsorption, showing the characteristic of easy functionalization. For example, physical embedding method is used to load growth factors like VEGF.^131^ Typical representatives of natural polymer-based hydrogels include alginate, chitosan, HA, and gelatin.

Polysaccharide polymers have a structure similar to human ECM. After entering the human body, they can simulate the ECM microenvironment of bone tissue, which is conducive to cell proliferation. The molecular chain of alginate contains carboxyl groups, which can rapidly form ionic cross-linking with divalent cations, and achieve the sol–gel transition in a short time. This process does not require other stimuli, and the gelation conditions are mild, so it is suitable for the repair of deep bone defects in the body. The hydrogel network formed by alginate often has high porosity, which is conducive to the exchange of nutrients.^132^ Its degradation products are glucuronic acid and mannuronic acid, which can be absorbed and metabolized by the body. Gelatin belongs to protein-based hydrogels and is the hydrolysis product of collagen.^133^ Gelatin retains the arginine-glycine-aspartic acid (RGD) sequence of collagen, which can specifically bind to integrins on the cell surface, promoting the proliferation and adhesion of osteoblasts.^134^ On the other hand, the sol–gel transition temperature of gelatin is close to human body temperature. After being injected into the body, it can gel automatically when encountering body temperature. This material is often used for the repair of small non-load-bearing bone defects such as alveolar bone and cranial bone. Bastami *et al.* developed a hydrogel composed of alginate, gelatin, and freeze-dried bone allograft nanoparticles, and then natural polymer-based hydrogels were used to directly introduce rat bone marrow mesenchymal stem cells (rBMSCs) to construct a composite hydrogel.^135^ They conducted experiments on a rat cranial defect model, and the results showed that this hydrogel can significantly enhance cell adhesion, proliferation, and osteogenic differentiation.

Natural polymer-based hydrogels often exhibit low mechanical strength and difficult-to-control degradation rates, which limits their application in repairing load-bearing bone defects that require long-term structural support.^136^ Among them, alginate degrades rapidly in the body, making it difficult to support the long-term needs of bone regeneration, while the degradation cycle of silk fibroin lasts for several months, which may lead to the problem of foreign body residue.^137,138^

### 3.2 Synthetic polymer

A primary characteristic of synthetic polymer-based hydrogels is controllability. As bone repair materials, synthetic polymer-based hydrogels need to fully meet the needs of bone regeneration. Artificially synthesized hydrogels allow for facile adjustment of parameters such as monomer type, cross-linking degree, and molecular chain topology, precisely customize mechanical properties, and obtain hydrogels with different properties to adapt to the physiological needs of different stages of bone repair.

**Fig. 8:**
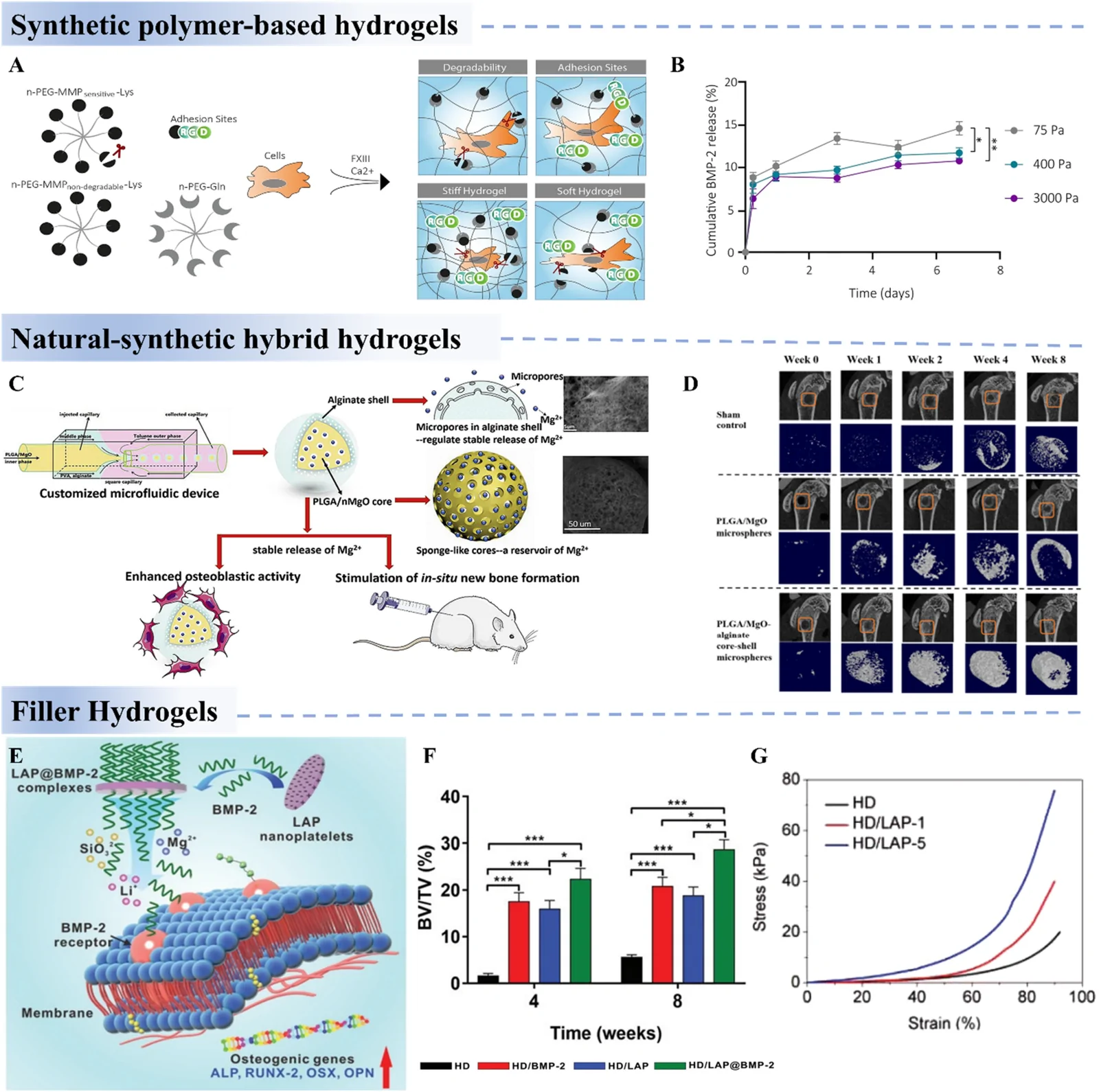
Schematic illustration of different hydrogel types for bone repair applications: (A) modular TG-PEG hydrogels tuning degradability, adhesion, and stiffness. (B) Cumulative BMP-2 release from TG-PEG hydrogels of varying stiffness, measured by ELISA (*n* = 4). (C) Microfluidic PLGA/MgO-alginate microsphere fabrication and osteogenesis. (D) Images and 3D reconstruction of lateral epicondyle in sham, PLGA/MgO, and PLGA/MgO – sodium alginate microsphere groups at different postoperative time points. (E) LAP@BMP-2 osteogenic mechanism. (F) BV/TV analysis. (G) Stress–strain curves. (A and B) Reproduced with permission. Copyright 2024, Springer Nature.^144^ (C and D) Reproduced with permission. Copyright 2018, Elsevier Ltd.^149^ (E–G) Reproduced with permission. Copyright 2020, Royal Society of Chemistry.^155^

Changing molecular chain length, block ratio, and functional group density is used to change the mechanical properties of synthetic polymers to adapt to various bone defect situations. Taking PEGDA hydrogel as an example, when the PEG concentration is appropriately increased, the molecular chain length increases, and the entanglement degree between molecular chains also increases. The pore size of the 3D network formed after cross-linking decreases, which can significantly enhance the compressive modulus of the material.^139,140^ The PCLA–PEG–PCLA block copolymer hydrogel regulates mechanical properties by changing the ratio of PCLA to PEG. Among them, PCLA as a rigid segment can provide mechanical support, and PEG as a flexible segment provides material elasticity. The ratio of the two allows for precise modulation based on application needs. The degradation rate of synthetic polymers is determined by monomer composition and cross-linking method. For example, in PEG–PCL–PEG block copolymer hydrogels, the PEG chain is hydrophilic and can accelerate the penetration of surrounding water, promoting the ester bond hydrolysis of the PCL chain. If the molecular weight of the PEG chain is increased, the degradation rate of the material can be improved.^141^ The cross-linked network formed by physical cross-linking is looser, and the resistance to water penetration is small, so physical cross-linking often has a slower degradation rate than chemical cross-linking.^142^ In the synthesis process of synthetic polymer-based hydrogels, introducing pH-sensitive carboxyl groups and enzyme-sensitive peptide segments can control the degradation of hydrogel materials in the defect microenvironment and achieve spatiotemporal controllable release of growth factors. Different functional groups can also be introduced to realize multifunctional integration of materials. For example, grafting RGD peptide segments can improve cell adhesion, increasing the spreading area of MC3T3 osteoblasts by 3 times. Loading growth factors such as BMP-2 and VEGF can promote bone regeneration and vascularization; introducing quaternary ammonium salts or silver nanoparticles can also inhibit the growth of *Staphylococcus aureus* and *Escherichia coli*, reducing the risk of postoperative infection. Wei *et al.* prepared a three-dimensional 3D porous scaffold of HA/PLGA. HA microspheres were prepared by spray-drying method, and then HA microspheres with different contents were mixed with PLGA.^143^ After the incorporation of HA microspheres into PLGA, the compressive strength of the scaffold was greatly increased, and the adhesion, proliferation, and osteogenic differentiation of bone marrow stem cells cultured on the scaffold surface were improved. Another study was based on the PEG system, fully exploring the flexibility of synthetic polymers in structural design.^144^ By precisely regulating mechanical stiffness, degradation rate, and introducing RGD cell adhesion peptides, a microenvironment suitable for cell proliferation and spreading was successfully constructed (Fig. 8A). In addition, this study endowed the hydrogel with bioactive factor delivery function, achieved the sustained release of BMP-2 growth factor, and effectively induced the osteogenic differentiation of the encapsulated progenitor cells (Fig. 8B). *In vivo* experiments further confirmed that by optimizing the physical properties of the hydrogel, cell migration and bone regeneration can be significantly promoted. This progress highlights the advantages of synthetic polymer-based hydrogels in integrating multifunctions such as physical support, biological adhesion, and drug delivery.

However, synthetic polymer materials also have existing challenges. Pure synthetic polymers lack cell recognition sites and have insufficient bioactivity, and need to be modified with biological molecules such as RGD peptide segments or collagen. For example, materials like PLGA produce acidic products during degradation, which may lead to a decrease in local pH value and trigger an inflammatory response.^145^ Concurrently, their scaled-up preparation process is relatively complex, and quality control is difficult. At present, only a few related products have entered the clinical application stage.

### 3.3 Natural–synthetic polymer matrices

Natural–synthetic hybrid hydrogels use physical and chemical methods to cross-link or form interpenetrating networks (IPN) between natural polymers (such as polysaccharides, proteins) and synthetic polymers (such as PEG, PLGA), forming a composite with both biocompatibility and mechanical properties.^146^ The favorable biocompatibility and cell recognition sites of natural polymers can promote cell adhesion, proliferation, and differentiation; synthetic polymers can provide a stable 3D network structure for hydrogels, protecting natural polymers from rapid degradation and extending their action time in the body. In terms of performance customizability, the designability of synthetic polymers makes the mechanical properties, degradation rate, and functionalization degree of composite hydrogels can be precisely regulated.^124^

There are many types of natural–synthetic hybrid hydrogels, and many typical representative materials show promising application prospects in the biomedical field. For example, chitosan–PEG composite hydrogel is a chemically cross-linked hydrogel formed by connecting the amino group of chitosan with the carboxyl group of PEG through an amide bond. It combines the biocompatibility and antibacterial properties of chitosan with the low immunogenicity and high stability of PEG, and has a wide range of applications in tissue engineering and drug delivery.^147^ Natural polymer–PLGA composite hydrogels are usually made of drug-loaded PLGA microspheres, and then the microspheres are combined with natural hydrogels. It combines the cell affinity and tissue regeneration-promoting ability of natural polymers with the controllable degradability and mechanical properties of PLGA. This composite hydrogel performs well in the fields of bone repair and skin wound repair.^148^ Lin *et al.* used a customized microfluidic capillary device to design a monodisperse core–shell microsphere delivery hydrogel composite system (Fig. 8C).^149^ The system is composed of PLGA biopolymer, alginate hydrogel, and magnesium oxide nanoparticles. The PLGA–MgO spongy spherical core loads Mg^2+^, while the alginate shell has an adjustable surface microporous network to accurately control the outflow of Mg^2+^. The novel core–shell microsphere system can effectively enhance the activity of osteoblasts *in vitro*. The hydrogel reduces the outflow of about 50 ppm of Mg^2+^*in vitro* for two weeks, and the concentration of 50–200 ppm of Mg^2+^ effectively promotes the proliferation and differentiation of pre-osteoblasts. The Young's modulus of the regenerated bone tissue by PLGA–MgO alginate microspheres has recovered about 96% of the damaged tissue, while the PLGA and PLGA–MgO groups induced 65% and 71% of bone modulus respectively (Fig. 8D).

However, in terms of preparation process, the preparation process of natural–synthetic hybrid hydrogels is usually relatively complex.^150^ It is necessary to precisely control reaction conditions such as temperature, pH value, and cross-linker concentration, otherwise it may lead to unstable performance of hydrogels. Some preparation methods may also use toxic and harmful chemical reagents such as cross-linkers and initiators. The residues of these reagents may affect the biological safety of hydrogels.^151^ For example, when using glutaraldehyde as a cross-linker to prepare chitosan–PEG composite hydrogel, the residue of glutaraldehyde may cause cytotoxicity and immune response.

### 3.4 Filler hydrogels

Filler hydrogels are novel functional materials constructed by combining inorganic nanoparticles with polymer matrices. Traditional polymer hydrogels are mostly soft materials at the kPa level, which are difficult to meet the needs of load-bearing tissue repair or flexible electronic devices. Inorganic-reinforced hydrogels use physical filling or chemical cross-linking methods to combine inorganic nanoparticles (such as hydroxyapatite, silica) with hydrogels, and can form a synergistic reinforcement effect with the polymer network, increasing the compressive modulus of hydrogels to the MPa level.^152^ The introduction of the inorganic phase also endows hydrogels with multifunctional integration capabilities. Different types of inorganic nanoparticles can bring additional properties to hydrogels. For example, bioactive inorganic particles such as hydroxyapatite and BG can promote bone cell adhesion and osteogenic differentiation, and perform well in the field of bone repair.^153^ Conductive nanoparticles such as graphene and CNTs can endow hydrogels excellent conductivity, which can be used for flexible electronic devices.^154^ Zhang *et al.* developed polysaccharide/LAPONITE®@BMP-2 (HD/LAP@BMP-2) hydrogel (Fig. 8E).^155^ Among them, LAPONITE® (LAP) nanosheets belong to an inorganic reinforcing phase, which can accelerate the gelation process through hydrogen bonding with the polysaccharide matrix, endowing the hydrogel with excellent mechanical properties and rheological behavior, as well as better injectability and self-healing ability (Fig. 8F and G). Interestingly, there is a strong static binding between LAP nanosheets and BMP-2, which can form a stable LAP@BMP-2 complex. The results show that these complexes can effectively maintain the intrinsic biological activity of BMP-2 and extend the release period to more than four weeks. Compared with using LAP or BMP-2 alone, the hydrogel incorporated with LAP@BMP-2 complexes can synergistically promote cell spreading, proliferation activity, and osteogenesis *in vitro* and *in vivo*. This non-invasive protein delivery system achieves sustained release of proteins and is expected to improve current orthopedic treatment strategies.

Filler hydrogels also have their limitations. Inorganic nanoparticles themselves have high surface energy and are prone to agglomerate into clusters in the hydrogel system, making it impossible to disperse uniformly in the polymer matrix. This will lead to non-uniform internal structure of the material, which not merely weakens the reinforcement effect of inorganic particles on hydrogels but also causes large fluctuations in the mechanical properties of the material. Inorganic-reinforced hydrogels also have the problem of poor interfacial compatibility. Inorganic particles and polymer matrices exhibit markedly different chemical structures and physical properties, resulting in weak interfacial bonding between them. Unmodified nano-hydroxyapatite tends to agglomerate within hydrogel matrices due to poor dispersion, forming surface cracks that compromise the mechanical integrity of the composites. Moreover, the weak interfacial adhesion of unmodified particles may lead to their detachment during scaffold biodegradation, potentially triggering adverse tissue responses.^127,128^ Biosafety is also a critical concern that cannot be ignored. If the particle size of some inorganic nanoparticles is not controlled properly or if the concentration is excessive, cytotoxicity may occur. For example, the toxicity of silver nanoparticles shows a clear concentration dependence. In the range of 10–50 µg mL^−1^, the survival rate of various cell lines (including macrophages and hepatocytes) decreases linearly with the increase of concentration.^156^

## 4. Research progress on the application of injectable hydrogels in bone repair

### 4.1 Critical-sized defect (CSD)

Critical-sized defect (CSD) refers to the smallest bone defect that cannot heal through its own repair mechanism, and the specific critical size varies for different sites. In various experimental studies, the rat cranial defect model (usually diameter ≥8 mm) is widely recognized as the standard CSD model for evaluating the effectiveness of bone repair materials due to its flat anatomical structure, simple operation, and low spontaneous healing tendency.^157^ Injectable hydrogels can form *in situ* and perfectly fill irregular CSD areas, making them ideal carriers. To address pivotal scientific issues in CSD repair such as poor microenvironment, lack of bioactivity, and insufficient mechanical support, existing research has shifted from single physical filling to multifunctional synergistic intervention. Through specific strategies like loading growth factors to reshape the osteogenic microenvironment, activating osteogenic induction, and introducing inorganic reinforcement phases to construct high-strength skeletons, substantial breakthroughs have been made in achieving complete bone tissue bridging and accelerating healing of large-segment defects.

#### 4.1.1 Alleviating impaired blood supply and inflammatory microenvironment in CSD

In the complex pathological process of CSD, severe trauma often leads to disruption of the local vascular network, triggering an ischemic and hypoxic microenvironment, which is the primary factor hindering the initiation of bone regeneration. Concurrently, the excessive inflammatory response in the defect area in the early stage cannot subside spontaneously. A large number of accumulated M1-type macrophages continuously secrete pro-inflammatory factors, leading to tissue fibrosis encapsulation, so that the defect area remains in an inflammatory microenvironment for a long time and cannot initiate the repair program. This vicious cycle of blood supply deficiency and uncontrolled inflammation is one of the core reasons why CSD is difficult to self-heal.

**Fig. 9:**
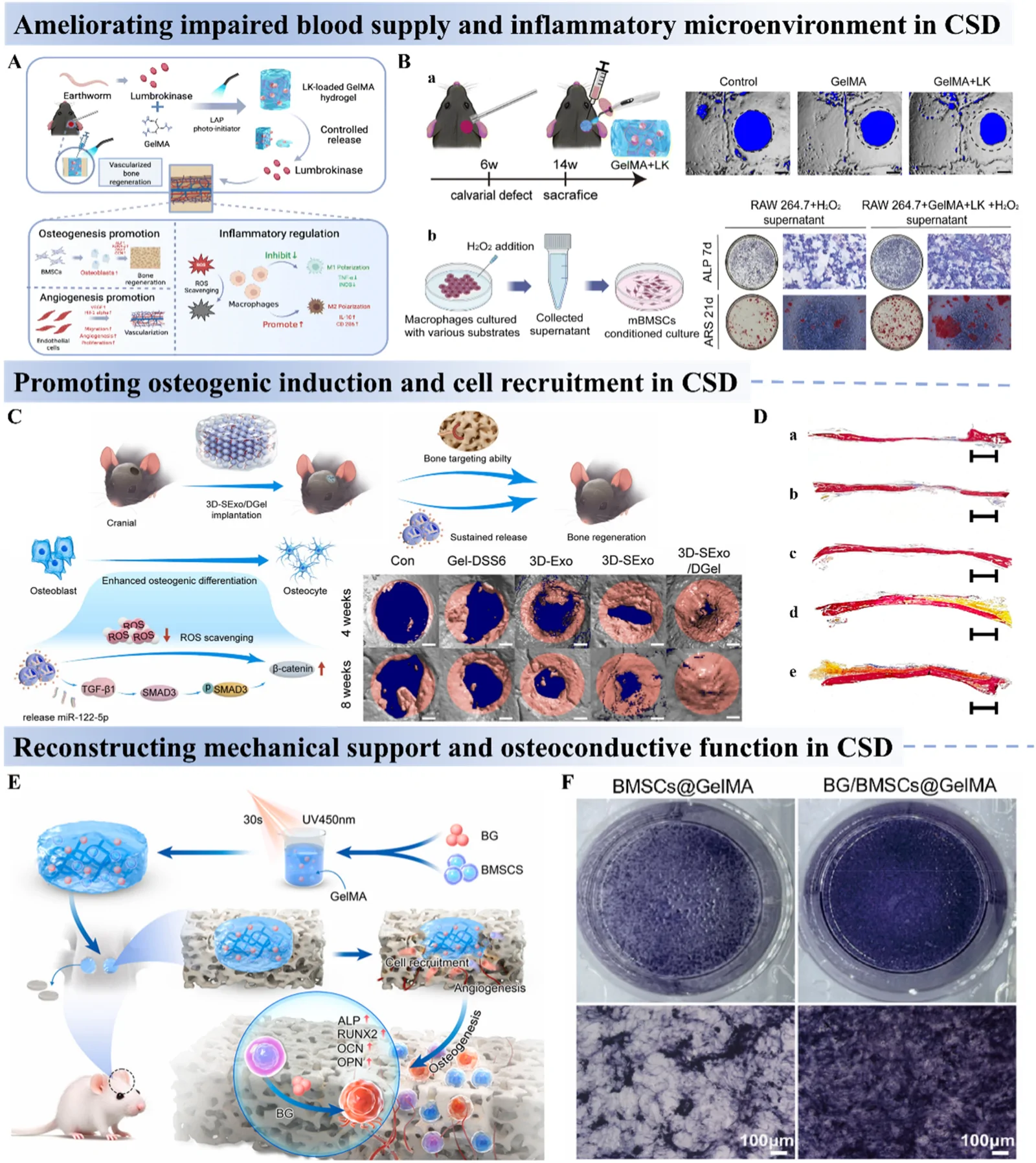
Schematic illustration of hydrogel repair of CSD: (A) LK-GelMA hydrogel for bone repair *via* sustained LK release, angiogenesis, osteogenesis, and immunomodulation. (B) (a) Cranial defect model and 8 weeks Micro-CT (1 mm). (b) RAW264.7-conditioned mBMSCs; ALP/ARS staining (scale bar, 50 µm, *n* = 3, *p < 0.05, *p* < 0.001). (C) Schematic illustration showing that 3D-SExo/DGel facilitates hypoxia-induced osteogenic differentiation of BMSCs *via* the Wnt/β-catenin pathway. *In vivo* calvarial repair is verified by Micro-CT imaging (scale bar = 500 µm). (D) Histological sections of the defective area of the skull after staining with alizarin red S: (a) Control, (b) HCF hydrogel, (c) HCF hydrogel + BMP-2, (d) HCF hydrogel + pericytes, (e) HCF hydrogel + BMP-2 + pericytes. Scale bar is 1 mm. (E) BG/BMSCs@GelMA fabrication and mechanism. (F) ALP (day 7) and ARS (day 14) staining of co-cultured MSCs. (A and B) Reproduced with permission. Copyright 2025, Royal Society of Chemistry.^158^ (C) Reproduced with permission. Copyright 2025, Elsevier Ltd.^161^ (D) Reproduced with permission. Copyright 2024, MDPI AG.^162^ (E and F) Reproduced with permission. Copyright 2023, Elsevier Ltd.^163^

To address this pathological feature, researchers have developed injectable hydrogels with microenvironment-regulating functions to reshape the regeneration conditions. For example, Zhang *et al.* incorporated lumbrokinase (LK) (an earthworm extract) into injectable GelMA hydrogel to achieve controlled release of LK, thereby enhancing vascularized bone regeneration in critical-sized defects (Fig. 9A).^158^ Notably, LK exhibited novel anti-inflammatory properties along with a dual capacity to promote both osteogenesis and angiogenesis (Fig. 9B). The LK-loaded GelMA hydrogel showed excellent biocompatibility, superior injectability and viscoelasticity, adjustable degradation kinetics, and optimal swelling properties. Comprehensive *in vitro* and *in vivo* evaluations confirmed the hydrogel's effective LK loading and sustained release characteristics. Some researchers used dimethyloxalylglycine (DMOG) to competitively bind the active site of prolyl hydroxylase (PHD), and deferoxamine (DFO) to chelate intracellular divalent iron ions (an essential cofactor of PHD enzyme) to indirectly inhibit PHD enzyme activity, thereby stabilizing and accumulating HIF-1α to mediate angiogenesis, effectively improving the ischemic state.^80,159^ For inflammation regulation, magnesium-loaded hydrogels have been proven to reverse the inflammatory environment that hinders healing by releasing hydrogen to scavenge ROS, inhibiting the IκB/NF-κB pathway to induce M2 macrophage polarization, while promoting osteogenesis and inhibiting osteoclastogenesis.^160^

#### 4.1.2 Promoting osteogenic induction and cell recruitment in CSD

CSD means that the defect size exceeds the blood supply and cell recruitment capabilities of the periosteum and surrounding tissues, leading to a lack of sufficient seed cells and osteogenic signal molecules in the central area of the defect. In the absence of exogenous intervention, the few remaining cells in the defect area are difficult to cross the huge repair distance, and the endogenous osteogenic signals are weak, making it impossible to initiate an effective callus formation response, ultimately leading to non-union or fibrous healing. This is the direct manifestation of CSD repair failure at the cellular and molecular levels.

To overcome the problem of insufficient active substances in CSD, injectable hydrogels are widely used as intelligent delivery carriers for growth factors and seed cells. Xiang *et al.* developed cultured exosomes modified with superparamagnetic iron oxide nanoparticles (SPION) (3D-SExo), and after encapsulating 3D-SExo in GelMA modified with bone-targeting peptide (Gel-DSS6), a composite hydrogel (3D-SExo/DGel) was obtained.^161^ This hydrogel can extend the retention time of exosomes, thereby enhancing bone repair ability. In addition, 3D-cultured exosomes are rich in miR-122-5p, which can promote osteogenic differentiation by activating the Wnt/β-catenin pathway (Fig. 9C). By combining mesenchymal stem cell-derived exosomes with such an intelligent delivery system, the Wnt/β-catenin signaling cascade is effectively triggered. Consequently, the local osteogenic potential is unlocked, enabling successful cell-free regeneration of critical-sized defects (CSD). Another study designed a heparin-conjugated fibrin (HCF) hydrogel containing BMP-2 and adipose-derived pericytes (ADPs).^162^ The HCF hydrogel can effectively control the release of BMP-2, and the eluted BMP-2 can significantly induce the osteogenic differentiation of ADPs, showing remarkable regenerative repair effects in the rat cranial critical-sized defect model (Fig. 9D).

#### 4.1.3 Reconstructing mechanical support and osteoconductive function in CSD

As a hard supporting tissue, bone tissue requires scaffold materials to provide sufficient mechanical strength during its repair process to maintain the stability of the defect space. However, traditional injectable hydrogels often have weak mechanical properties and fast degradation rates. In CSD sites bearing physiological loads, insufficient mechanical strength can lead to scaffold collapse or deformation, failing to provide an effective physical space for new bone growth. Moreover, rapid degradation may cause the scaffold to disappear before bone tissue matures, resulting in repair interruption or secondary defects. This is an inherent defect of soft hydrogel materials when applied to bone repair.

To address the deficiencies in mechanical support and osteoconductivity, inorganic nano-composite reinforcement has been employed to enhance mechanical strength, while structural design (*e.g.*, porous scaffolds) focuses on improving osteoconductivity integrity. Ai *et al.* combined BG, BMSCs, and GelMA (Fig. 9E).^163^ Among them, BG can release calcium, phosphorus and other ions to promote local osteoblast differentiation and mineralization; BMSCs can differentiate into osteoblasts and vascular endothelial cells, participating in new bone formation and vascular network construction; GelMA serves as a cell carrier and 3D supporting structure, providing a suitable microenvironment for cells. The synergistic effect of the three significantly accelerated the vascularization process in the defect area, providing sufficient blood supply for bone regeneration and effectively promoting the formation and maturation of new bone tissue (Fig. 9F). The composite scaffold constructed by photopolymerization can greatly improve the compressive modulus of the material. The 6BG/BMSCs@GelMA hydrogel injected into critical-sized cranial defects further confirmed its excellent angiogenic and bone-regenerative abilities. In addition, self-healing hydrogels constructed based on dynamic/covalent dual-cross-linking structures have also been successfully applied to CSD repair. They can rapidly repair the network structure after being damaged by external forces, maintaining the long-term mechanical integrity of the defect area and ensuring the continuity and stability of the bone regeneration process.^164,165^

### 4.2 Load-bearing bone defect

Load-bearing bone defects refer to structural and functional abnormalities such as bone cracks, fractures, and bone contusions in the main load-bearing bones of the human body (such as the femur, tibia, calcaneus, vertebral bodies, *etc.*) caused by external force impacts, long-term overuse, or pathological factors during standing and walking. Acute injuries are often accompanied by pain, swelling, limited activity, and even deformity; chronic overuse manifests as recurrent dull pain, and the symptoms worsen after exertion. In the human skeletal system, load-bearing bones like the femur and tibia need to bear the body weight and multiple mechanical stimuli such as impact, bending, and torsional forces generated during movement for a long time. Therefore, for hydrogels repairing load-bearing bone defects, in addition to having favorable biocompatibility and bone regeneration, it is more important to have sufficient mechanical strength to withstand various complex loads in daily activities.^146^ In terms of performance, the material must simulate the mechanical properties of natural bone to ensure stable support for the bone structure after implantation and promote the regeneration and integration of bone tissue.

#### 4.2.1 Repair of femoral defects

**Fig. 10:**
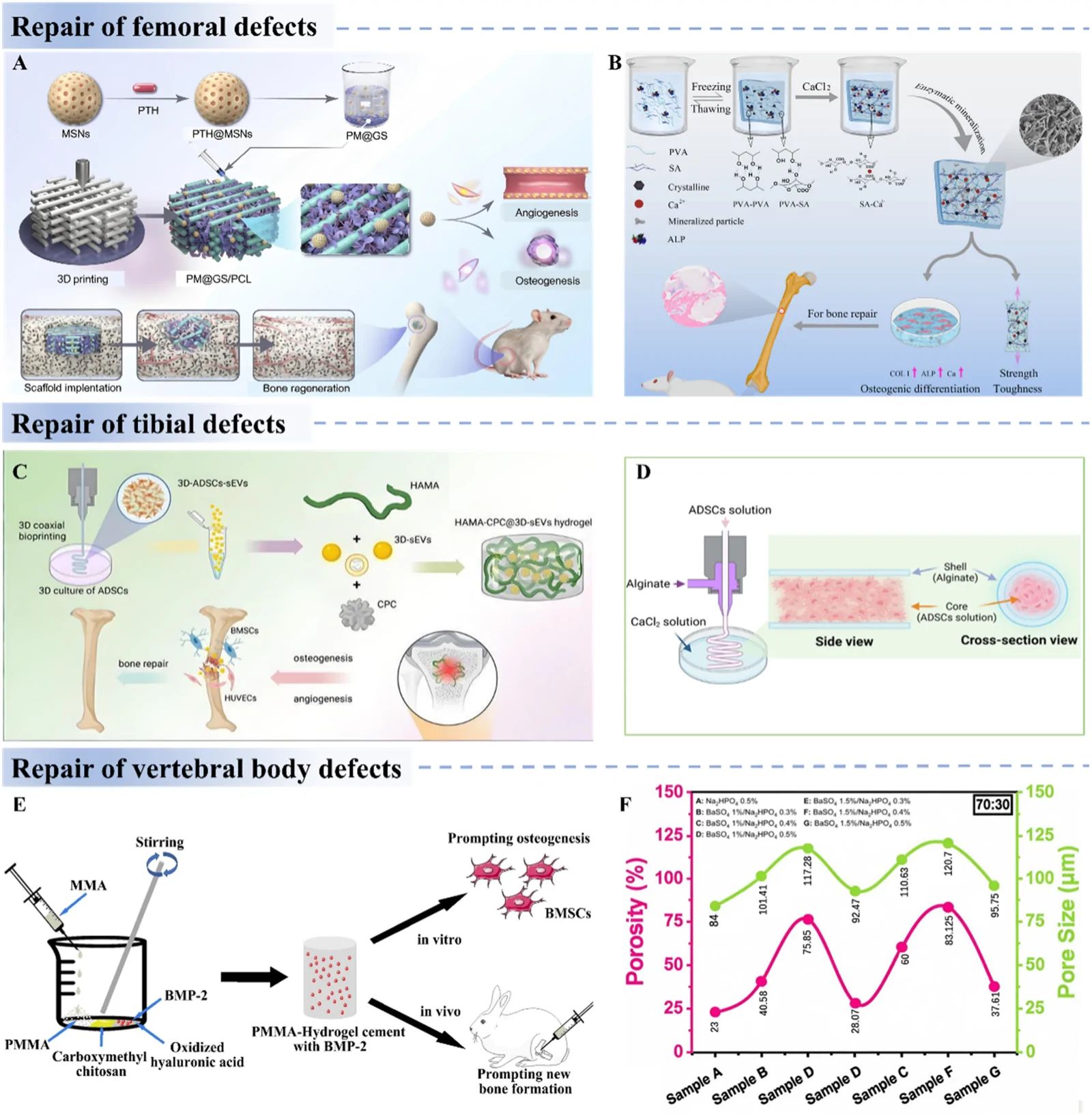
Schematic illustration of hydrogel and scaffold strategies for repairing femoral, tibial, and vertebral body defects: (A) schematic of PM@GS/PCL scaffold construction and rat femoral bone regeneration. (B) Schematic illustration of hydrogel fabrication, mechanical and biological properties, and applications in bone repair. (C) Study design schematic of HAMA-CPC@3D-sEVs for rat bone regeneration. (D) Schematic diagram of cellular microfiber structure fabricated based on coaxial 3D bioprinting. (E) PMMA-hydrogel hybrid cement fabrication for *in vitro* and vivo analyses. (F) Porosity and pore size of 70 : 30 ALG/PVA/BaSO_4_ hydrogels (peak porosity 85.89% with 1.5% BaSO_4_/0.4% Na_2_HPO_4_). (A) Reproduced with permission. Copyright 2024, Changchun Institute of Applied Chemistry, Chinese Academy of Sciences.^166^ (B) Reproduced with permission. Copyright 2024, American Chemical Society.^167^ (C and D) Reproduced with permission. Copyright 2025, BMC.^168^ (E) Reproduced with permission. Copyright 2022, Springer Singapore.^172^ (F) Reproduced with permission. Copyright 2025, Royal Society of Chemistry.^173^

The femur is the longest and strongest bone in the human body, bearing the largest axial load and dynamic shear stress. The repair of femoral defects often involves large-segment bone loss, which poses strict tests on the fatigue resistance, load-bearing capacity, and bone integration speed of the repair materials. Large-segment femoral defects are often accompanied by severe soft tissue damage and blood supply disruption, so the functional design of hydrogels and the synergistic effect of biological induction have received great attention. Traditional pure hydrogel materials are difficult to withstand the huge stress in the femur, while pure rigid implants have the problem of slow healing. To this end, researchers such as Yang *et al.* constructed a PM@GS/PCL integrated scaffold and used it in a rat femoral defect model (Fig. 10A).^166^ By loading PTH nanoparticles, the scaffold had strong biological induction activity, which could significantly promote angiogenesis and osteogenic differentiation. Furthermore, the 3D-printed PCL skeleton ensured the load-bearing requirements of the femur. *In vivo* experiments in the rat femoral defect model confirmed that it effectively accelerated the bone integration and repair process.

On the other hand, overcoming the mechanical limitations of hydrogels is the key to repairing large-segment load-bearing bones. Zhang *et al.* designed an enzymatically mineralized PVASA double-network hydrogel (Fig. 10B).^167^ The study used polyvinyl alcohol (PVA) and sodium alginate (SA) to form a double-network structure through repeated freeze-thawing and ionic cross-linking, and combined enzymatic mineralization to achieve synergistic improvement of mechanical and biological properties. The prepared hydrogel had a Young's modulus of 1.03 MPa, a storage modulus of 103 kPa, and a toughness of 1.86 MJ m^−3^, and remained highly tough without brittleness after mineralization. In the rat femoral defect model, this hydrogel effectively promoted new bone formation.

#### 4.2.2 Repair of tibial defects

The tibia is the most important load-bearing diaphysis in the lower leg. Severe damage to the local blood supply after tibial defects will lead to abnormally slow bone regeneration, which becomes another significant bottleneck restricting the repair effect. To overcome this biological problem, researchers combined engineered cell extracellular vesicles with bioactive hydrogels. Xu *et al.* constructed an injectable hyaluronic acid methacryloyl-calcium phosphate cement (HAMA-CPC) hydrogel system (HAMA-CPC@3D-sEV) that integrates high-yield 3D bioprinted adipose-derived stem cell small extracellular vesicles (Fig. 10C and D).^168^*In vitro* experiments confirmed that the functionalized hydrogel could significantly promote the proliferation, migration, and osteogenic differentiation of bone marrow stromal cells, and effectively induce angiogenesis of human umbilical vein endothelial cells. In the *in vivo* evaluation of the rat tibial defect model, this therapeutic strategy showed excellent bone regeneration potential, and the bone volume fraction (BV/TV), bone volume (BV), and trabecular thickness were significantly increased at four weeks after surgery. In-depth mechanism studies showed that 3D-bioprinted adipose-derived stem cell small extracellular vesicles (3D-sEVs) upregulated the expression of S1PR1 in endothelial cells by delivering highly expressed nicotinamide phosphoribosyltransferase (NAMPT) protein, thereby promoting VEGF expression and angiogenesis.

The tibia needs to bear most of the axial load of the human body. After the defect occurs, the discontinuity of the bone structure leads to the blockage of the mechanical conduction path. If the repair material lacks sufficient strength and stiffness, it is easy to collapse or deform under load, leading to repair failure. To solve this mechanical problem, Wang *et al.* designed and prepared a bone repair scaffold with excellent mechanical load-bearing capacity.^169^ The study used fused deposition modeling (FDM) 3D printing technology to construct a rigid skeleton (PCL@BG20) using PCL as the matrix and BG as the inorganic reinforcement phase (20%, w/w), and then filled the pores with GelMA hydrogel loaded with stromal cell-derived factor 1α (SDF-1α). This construction strategy fully exerted the excellent mechanical properties and potent osteogenic activity of BG, and synergized with the biological function of SDF-1α to induce the migration of mesenchymal stem cells and vascular endothelial cells.^170^ The mechanical test results showed that the compressive strength of the PCL@BG20-GelMA/SDF-1α composite scaffold was as high as 44.89 ± 3.45 MPa, which matched the mechanical properties of natural cancellous bone and could provide stable support for the defect site. *In vivo* experiments further confirmed that the scaffold showed excellent performance in the repair of proximal tibial defects in rabbits. Its mechanical strength effectively resisted the load of the lower limb, and the synergistic effect of SDF-1α and BG significantly promoted vascularized bone regeneration, achieving the dual goals of structural support and functional reconstruction. In addition, existing research has further explored the strategy of synergistically enhancing mechanical properties and biological functions through nanocomposites to promote bone regeneration. The study developed a calcium/tannic acid-reduced graphene oxide/gelatin methacryloyl (CTAG/GelMA) nanocomposite hydrogel, which used GO to significantly enhance the mechanical properties of the scaffold, and regulated the immune microenvironment through the synergistic effect of Ca^2+^ and tannic acid-reduced graphene oxide (TA-rGO). This hydrogel provided stable mechanical support while inducing M2 polarization of macrophages and promoting angiogenesis, achieving efficient synergy between mechanical enhancement and bioactivity, and significantly accelerating bone regeneration.^171^

#### 4.2.3 Repair of vertebral body defects

Vertebral body defects are mostly caused by spinal tumors, burst fractures, or osteoporotic compression fractures. The core of repair lies in restoring vertebral height, maintaining spinal stability, and preventing vertebral collapse. Percutaneous vertebroplasty (PVP) and percutaneous kyphoplasty (PKP) are the mainstream clinical methods for treating vertebral defects at present, and the traditional filling material is polymethyl methacrylate (PMMA) bone cement. However, PMMA has defects such as non-degradability, exothermic damage, and excessive mechanical properties leading to fractures of adjacent vertebrae. Therefore, degradable hydrogel bone adhesives with adjustable mechanical properties have become a research focus. In this context, Sun *et al.* successfully developed a PMMA-hydrogel mixed cement loaded with BMP-2 to improve the osteogenic effect of percutaneous vertebroplasty and kyphoplasty, focusing on solving the problems of degradability and mechanical matching of traditional bone cement (Fig. 10E).^172^ Introducing the hydrogel component significantly improved the material's biodegradability, overcoming the limitation of non-degradable traditional PMMA which hinders bone regeneration. The mechanical properties of the material were optimized and reorganized, providing sufficient compressive strength to support the vertebrae while avoiding the stress shielding effect caused by excessively high modulus, better matching the mechanical environment of vertebral cancellous bone, and realizing the synergistic effect of appropriate mechanical properties and favorable biodegradability. Combined with the slow-release osteogenic effect of BMP-2, this material significantly promoted the repair and regeneration of bone defects.

In the research field of nucleus pulposus regeneration, important progress has been made in the performance optimization of injectable scaffolds. Researchers studied a new method of introducing Na_2_HPO_4_ and BaSO_4_ into a single system at the same time and successfully screened the optimal ALG/PVA hydrogel formula with appropriate gel time, excellent biocompatibility, and imaging function.^173^ This strategy innovatively solved the requirements of gel kinetics and imaging for injectable NP scaffolds (Fig. 10F). Although the compressive strength of the system has not yet fully met the physiological load requirements, it shows significant advantages in maintaining the hydration environment of the intervertebral space and supporting cell activity.^173^

### 4.3 Nonunion and delayed fracture healing

Nonunion and delayed fracture healing refer to conditions where bone healing fails to occur or progresses beyond the normal timeframe following a fracture.^174^ Traumatic nonunion and delayed fracture healing are mainly due to excessive soft tissue stripping during fracture, which damages the blood supply and nutrient supply around the fracture site. Vascular injury leads to insufficient blood supply at the fracture site, resulting in hypoxia and metabolic disorders of osteocytes, affecting callus formation; infected nonunion and delayed fracture healing are caused by local infection after fracture.^175^ The inflammatory response affects the blood supply and bone regeneration at the fracture site, destroys bone tissue, and interferes with the conversion process of callus, leading to delayed or non-healing of fracture.^176^ These two complications seriously hinder the structural reconstruction and functional recovery of bones. The core cause is mainly the destruction of the local microenvironment at the fracture site. Whether it is vascular rupture caused by physical trauma or an inflammatory storm triggered by infectious factors, it will eventually cause local ischemia and hypoxia, thereby inhibiting osteogenic activity. Therefore, ameliorating the ischemic microenvironment has become a significant breakthrough in treatment. Researchers often load stem cells or pro-angiogenic factors into hydrogels to reconstruct the blood supply function of the trauma site, or use them as intelligent delivery platforms for antibacterial/anti-inflammatory drugs to eliminate infection, providing microenvironment regulation strategies for the treatment of nonunion.

#### 4.3.1 Traumatic nonunion and delayed fracture healing

Traumatic nonunion and delayed fracture healing are mainly due to excessive soft tissue stripping during fracture, which seriously damages the blood supply and nutrient supply around the fracture site. This vascular injury directly leads to insufficient blood supply at the fracture end, which in turn causes hypoxia and metabolic disorders of osteocytes, fundamentally hindering the normal formation of callus and making it impossible for the fracture to heal within the normal healing period.^175^

**Fig. 11:**
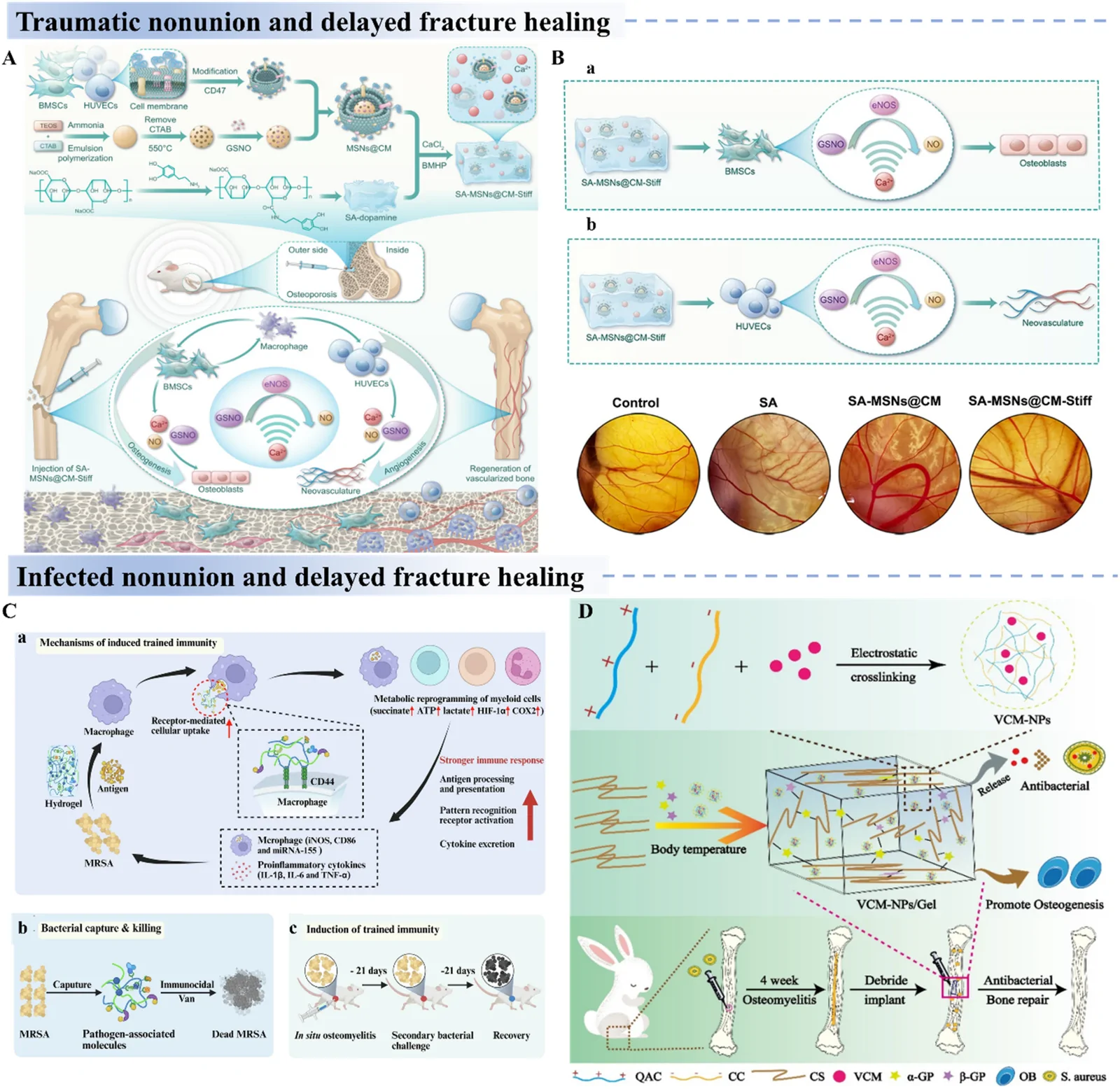
Schematic illustration of the design and mechanisms of hydrogel materials for the treatment of traumatic or infected nonunion and delayed fracture healing: (A) Biomimetic nanomaterial coupling osteogenesis/angiogenesis with immunomodulation. (B) (a) SA-MSNs@CM-Stiff promotes BMSC osteogenesis *via* NO/cGMP pathway. (b) SA-MSNs@CM-Stiff promotes HUVEC angiogenesis *via* NO/cGMP pathway and chick CAM assay (*n* = 3). (C) (a) Mechanisms of induced trained immunity by the hydrogel. (b) Bacterial capture and bactericidal properties of the hydrogel. (c) Induction of trained immunity and prevention of relapse by the hydrogel. (d) Schematic of VCM-NPs/Gel for osteomyelitis therapy. (A and B) Reproduced with permission. Copyright 2024, American Association for the Advancement of Science.^177^ (C) Reproduced with permission. Copyright 2026, Springer Nature.^179^ (D) Reproduced with permission. Copyright 2020, Dove Medical Press.^180^

To solve the local ischemia caused by trauma, the key to treatment lies in reconstructing local blood supply, improving the microenvironment for stem cell colonization, and promoting angiogenesis. Aiming at the significant problems of improving the osteogenic microenvironment and stem cell homing during bone regeneration, a novel biomimetic hydrogel system has been developed recently.^177^ This hydrogel, as a carrier of *S*-nitrosoglutathione and Ca^2+^, could induce bone marrow mesenchymal stem cells and human vascular endothelial cells to release bioactive nitric oxide (NO). By activating the NO/cyclic guanosine monophosphate signaling pathway, it effectively promotes the coupled process of osteogenesis and angiogenesis (Fig. 11A). Meanwhile, the system achieves active recruitment of endogenous bone marrow mesenchymal stem cells by conjugating the stem cell homing peptide SKPPGTSS. Using the rat distal femoral defect model, the biomimetic hydrogel significantly increased the formation of bone tissue and new blood vessels (Fig. 11B) and showed excellent immunomodulatory functions. Mousaei *et al.* first combined adipose-derived mesenchymal stem cells (Ad-MSCs), cancellous bone grafts (CBG), and chitosan hydrogel (CHI) to improve the healing of nonunion fractures.^178^ This hydrogel can significantly up-regulate VEGF gene expression, improve the local ischemic microenvironment to establish rat fracture nonunion, and at the same time induce an increase in BMP-2 expression to promote osteogenic differentiation. The results of animal models showed that positive effects on fracture treatment were produced as early as two weeks after surgery. In all groups receiving Ad-MSCs treatment, Ad-MSCs had positive effects on angiogenesis and osteogenic differentiation. The research results confirmed that the combined use of cancellous bone grafts and MSCs can more effectively treat nonunion.

#### 4.3.2 Infected nonunion and delayed fracture healing

Infected nonunion and delayed fracture healing are caused by local infection after fracture, and the inflammatory response seriously interferes with the normal physiological process at the fracture end. Infection not only directly damages bone tissue but also hinders the conversion and maturation of callus. Concurrently, vascular damage caused by inflammation further aggravates local ischemia and nutritional disorders, ultimately leading to delayed or even non-healing of fracture.^176^ To treat such complications, it is necessary to focus on solving the severe blood supply disorders caused by osteomyelitis on the basis of controlling infection.

Aiming at the difficult problem of repairing nonunion with accompanying osteomyelitis pathological features, Chen *et al.* developed an injectable gallium-copper-vancomycin & hyaluronic acid-aldehyde-bovine serum albumin (GaCuVan&HACHO-BSA) hydrogel, which can self-assemble *in situ* in the medullary cavity (Fig. 11C).^179^ It not only shows excellent pathogen clearance ability but more importantly, it captures bacteria and virulence factors through multivalent interactions, activating “trained immunity” based on metabolic reprogramming. While efficiently clearing pathogens, it induces long-term immune memory to prevent recurrence and synergizes with the osteogenic properties of glycyrrhizic acid to promote bone tissue regeneration. This strategy successfully integrates antibacterial defense, immunomodulation, and bone repair functions, providing an innovative solution for the clinical treatment of osteomyelitis and infected nonunion. Another study developed a local delivery system of chitosan-based vancomycin (VCM) -nanoparticles/thermosensitive hydrogel (Fig. 11D).^180^ The system significantly improves drug loading efficiency through charge adsorption technology, achieving long-acting sustained release of vancomycin and efficient clearance of *Staphylococcus aureus*. Animal experiments confirmed that this hydrogel could promote osteoblast proliferation and accelerate bone repair while effectively controlling osteomyelitis infection.

## 5. Current challenges and strategies

**Fig. 12:**
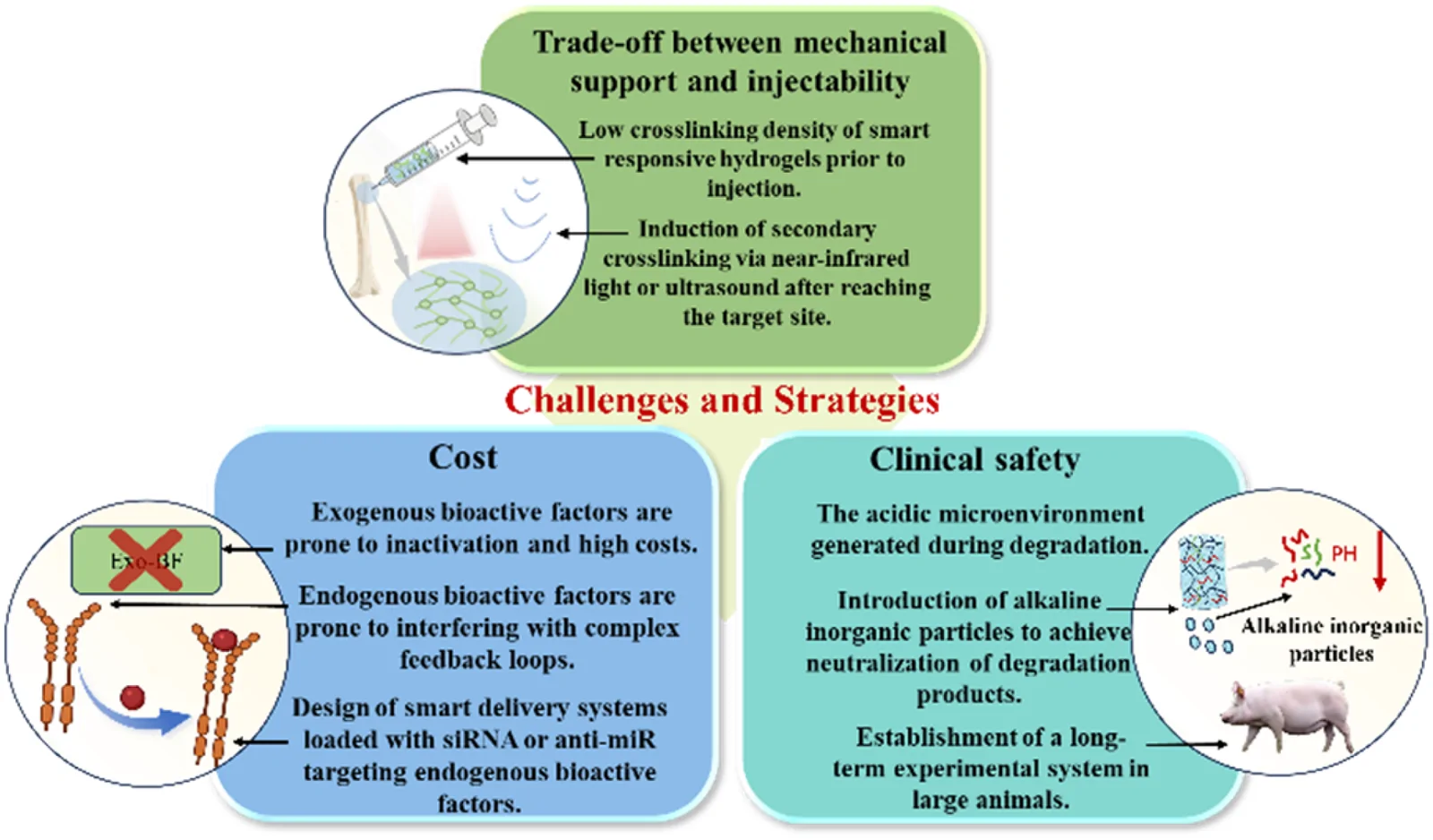
Current challenges and strategies for injectable hydrogels.

An ideal bone repair scaffold needs to simulate both the structural and functional properties of natural bone matrix, have clinical operational plasticity, and ensure long-term implant safety. At present, there is an inherent contradiction between the mechanical support strength and injectability of hydrogel systems. Exogenous growth factors are limited in application due to their high cost and poor stability, and the biocompatibility of material degradation products and the long-term efficacy evaluation are still insufficient. Here, we propose potential solutions to support the development of high-performance and high-safety bone repair hydrogel systems (Fig. 12).

### 5.1 Trade-off between mechanical support and injectability

In terms of material design, it is challenging for hydrogels to simultaneously meet sufficient mechanical support and injectability. For bone defects such as repairing load-bearing bones, hydrogel scaffolds are required to have higher compressive strength, tensile modulus, and fatigue resistance. To achieve these mechanical indicators, researchers introduce inorganic reinforcement phases into the materials. These phases can form entanglement and cross-linking with polymer chains through multivalent metal ions, hydrogen bonds, *etc.*, increasing the density of the hydrogel network and enhancing its rigidity and toughness. High cross-linking density improves the rheological properties of hydrogels, which directly leads to excessively high viscosity of hydrogels (>10^3^ Pa s), significantly increasing the resistance during the injection process, and even causing needle blockage due to excessive local pressure, affecting the smooth progress of clinical operations.^181,182^

To address the brittleness and limited degradability resulting from high cross-linking densities when advanced strategies are lacking, solutions are proposed here. A spatiotemporally separated cross-linking strategy is adopted to construct smart responsive hydrogels. That is, during the injection stage, the hydrogel material maintains a low cross-linking density or a single-network state to achieve smooth and fluent injection. After the material reaches the target site, specific responsive groups in the gel are activated by near-infrared light or ultrasound to induce secondary cross-linking or network reconstruction, and then a network structure with high mechanical performance is formed. In near-infrared light triggering, up-conversion nanoparticles or organic photothermal reagents convert light energy into local thermal energy, which can drive the cross-linking reaction of pre-designed thermosensitive hydrogels (such as the phase separation of poly(*N*-isopropylacrylamide) (PNIPAM)) or thermally activated click chemistry reactions (such as Diels–Alder addition). Ultrasound triggering relies on the mechanical force effect. For example, the local high temperature and high pressure generated when cavitation bubbles collapse can activate sensitive groups or drive the radical polymerization of sonosensitive cross-linking agents, ultimately achieving the responsive properties of hydrogels at specific spatiotemporal scales. These two triggering methods solve the contradiction between injection and mechanical performance in clinical practice and avoid chemical stimulation to surrounding tissues.

Beyond relying solely on post-injection solidification, an alternative pivotal strategy to resolve this trade-off lies in the ingenious design of network topology to regulate rheological behavior. Specifically, constructing dynamic physical cross-linking networks endows hydrogels with superior shear-thinning and thixotropic properties. Under the shear stress of injection, these reversible bonds—such as hydrogen bonds, ionic interactions, or host–guest assemblies—dissociate transiently to significantly reduce viscosity, facilitating smooth extrusion through narrow needles. Once the shear force is removed, the rapid re-association of these dynamic bonds ensures immediate recovery of the network structure. This “inject-and-recover” mechanism not merely minimizes the injection resistance for high-solid-content hydrogels but also effectively prevents post-injection leakage. Furthermore, the introduction of nano-fillers, such as silica nanoparticles or LAPONITE®, can modulate the yield-stress behavior of the composite. These systems maintain a solid-like state at rest to provide necessary mechanical support for bone defects but yield to flow like liquids when the applied stress exceeds a critical threshold, thereby perfectly balancing the conflicting requirements of structural rigidity and operational deliverability.

### 5.2 Cost

Injectable hydrogels face the challenge of high cost in the clinical translation of bone repair, and a large part of the reason comes from growth factors that regulate the microenvironment. Endogenous growth factors have the challenge of difficult regulation, so the strategy of loading exogenous recombinant growth factors into hydrogel systems and delivering them into the body has been widely used by researchers. The source of exogenous growth factors requires complex mammalian cell culture processes and strict cold chain storage and transportation requirements, resulting in significantly high production costs. In addition, constructing stable high-expression cell lines is time-consuming, taking about 12–18 months. For instance, the high cost of recombinant proteins such as BMP-2 poses a significant barrier to clinical translation; a single dose for spinal fusion surgery can reach several thousand US dollars, which greatly limits clinical application. Moreover, exogenous recombinant proteins are easily enzymatically degraded and conformationally unstable in the physiological environment and will inactivate in a short time.^183^ For example, the half-life of natural fibroblast growth factor-2 (FGF-2) is only two to four minutes.^184^ Jeong *et al.* reported that the half-life of recombinant human bone morphogenetic protein-2 (rh-BMP-2) was relatively short, about 7–16 minutes and 0.3 days, indicating that it was cleared quickly in the body, and endogenous BMP-2 was easily degraded by proteolysis, which further shortened the half-life of recombinant proteins.^185^ Most growth factors are rich in disulfide bonds and are sensitive to temperature, pH, and redox environment. Incorrect folding not merely leads to loss of activity but may also form immunogenic aggregates.^186^

Due to the problems of easy inactivation and high cost of exogenous bioactive factors, the solution can be focused on the regulation of endogenous active factors to solve the dilemma that exogenous bioactive factors cannot solve. The endogenous growth factor network has complex dynamics, and factors are regulated at multiple levels including transcription, post-transcription, translation, and post-translation, and there are complex feedback loops. For example, the TGF-β/activin signaling pathway can promote or inhibit different aspects of the regeneration process.^187^ The difficulty in regulating endogenous proteins is reflected in the over-expression of their inhibitors or the silencing of pivotal signaling pathways. To solve this problem, smart delivery systems loaded with specific small interfering RNA (siRNA) or anti-microRNA (anti-miR) can be designed. miR-214 is a typical negative regulator of osteogenesis.^188^ After over-expression, it targets and inhibits activating transcription factor 4 (ATF4), blocking the expression of osteogenic differentiation genes runt-related transcription factor 2 (RUNX2) and osteocalcin (OCN). By delivering antagomiR-214 (miR-214 inhibitor), the inhibition of ATF4 can be relieved, the BMP-2/Smad signaling pathway can be activated, and the expression of endogenous growth factors such as BMP-2 can also be up-regulated.^189^

### 5.3 Clinical safety

The biocompatibility of material degradation products has always been a concern. Some synthetic polymers such as PLGA produce acidic products like LA and GA during degradation, which may form an acidic microenvironment locally.^190^ This acidic microenvironment significantly inhibits alkaline phosphatase activity, leading to hindered new bone calcification.^191,192^ Alkaline phosphatase is an essential enzyme secreted by osteoblasts. It catalyzes the hydrolysis of phosphates during the bone mineralization process, providing necessary conditions for the formation of hydroxyapatite crystals. Its inhibition will directly delay the bone tissue repair process and even cause local inflammatory reactions and fibrous encapsulation.^193^ Another safety issue is that most research is limited to animal experiments, and there is a lack of long-term safety data for more than five years. In existing literature, most animal experiments have an observation period of less than 6 months, while clinical data shows that the complete remodeling cycle of human bone healing (such as nonunion) is usually far beyond this.^194–196^ This temporal discrepancy between preclinical animal models and clinical evaluation often contributes to translational bottlenecks, limiting the progression of materials that show promise in animal studies during phase II clinical trials.

For the acidic microenvironment generated during degradation, alkaline inorganic particles such as BG can be introduced into hydrogel materials to neutralize the degradation products. The silicate network of BG dissolves in the physiological environment and can release bioactive ions such as calcium ions and silicon ions.^197^ If the dissolution kinetics of BG are precisely tuned to match the degradation rate of the polymer, dynamic pH homeostasis of the material can be achieved.^198^ Moreover, the calcium hydroxide layer formed on its surface can also promote bone integration.^199^ In terms of hydrogel material evaluation, a long-term experimental system in large animals needs to be established. The bone anatomical structure, metabolic rate, and healing mechanism of animals such as pigs and sheep are highly similar to those of humans.^200^ Then, follow-up for one to two years can directly cover the complete degradation of the material, the maturation of bone remodeling, and potential delayed inflammatory risks.

## 6. Conclusions and perspective

Injectable hydrogels, with their core advantages of minimally invasive nature, biomimetic microenvironment remodeling, and functional plasticity, provide new strategies for bone defect repair that break through the traditional framework. This paper systematically sorts out the design strategies of hydrogel materials such as mechanical enhancement, bioactive functionalization, and degradation matching, and summarizes the classification of current hydrogel materials and their repair efficiency in different bone defect scenarios. However, in the process of moving towards true clinical translation, materials still face severe barriers such as the inherent contradiction between mechanics and injectability, the high cost and easy inactivation dilemma of introducing exogenous active components, while clinical translation is hindered by the lack of data on acidic degradation products and long-term safety.

The future development of bone repair hydrogels needs to break out of the limitation of single-performance optimization. Smart responsive groups should be designed to rely on degradation rate differences or stimulus-responsive gating to achieve on-demand release of different functional factors, temporal-sequential synergy and spatial matching, and precise regulation of complex microenvironments. For example, an active regulation system that can adaptively reconstruct with the spatiotemporal dynamics of bone healing should be constructed to fundamentally solve the contradiction that mechanical support and minimally invasive delivery are difficult to balance. Simultaneously, hydrogel materials should be designed to dynamically adjust their biological activity at different stages of bone healing. By precisely intervening in gene expression or the immune microenvironment, the regenerative mechanism of endogenous factors can be activated to achieve low-cost and long-lasting bone regeneration. Finally, to address the gap in clinical translation, future research needs to establish strict evaluation standards throughout the entire life cycle of materials, especially to fill the cognitive gap from short-term small animal models to long-term large animal and human real-world environments, ensuring the ultimate safety and effectiveness of materials in the body, and ultimately promoting bone repair treatment towards true personalization and precision. These innovative directions will be realized in a more complete standard system of mechanical properties, degradation kinetics, and bioactivity evaluation in the future, promoting the clinical translation of injectable hydrogels for bone repair.

## Abbreviations

Ac: Acrylic acid
ALN: Alendronate
ALP: Alkaline phosphatase
APEG: Poly(ethylene glycol) acrylate
Ad-MSCs: Adipose-derived mesenchymal stem cells
ADPs: Adipose-derived pericytes
ADSCs: Adipose-derived stem cells
anti-miR: anti-MicroRNA
ARS: Alizarin Red S
ATF4: Activating transcription factor 4
BBPs: Bone-binding peptides
BG: Bioactive glass
BMSCs: Bone marrow mesenchymal stem cells
BV: Bone volume
BV/TV: Bone volume fraction
Ca^2+^: Calcium ions
CBG: Cancellous bone grafts
CGFs: Concentrated growth factors
CHI: Chitosan hydrogel
CMC: Carboxymethyl chitosan
CNTs: Carbon nanotubes
CORMs: Carbon monoxide-releasing molecules
CPO: Calcium peroxide
CPH: Chitosan-polyvinyl alcohol
CPHH: HAp-coated CPH hydrogel
CS: Chitosan
CSD: Critical-sized defect
CTL: Thiolated chitosan-modified paclitaxel liposomes
CuAAC: Copper-catalyzed azide–alkyne cycloaddition
DNA: Deoxyribonucleic acid
ECM: Extracellular matrix
EGCG: Epigallocatechin gallate
ELRs: Elastin-like recombinant polymers
ETTMP: Ethoxylated trimethylolpropane tri(3-mercaptopropionate)
FGF-2: Fibroblast growth factor-2
FND-ZHD: Injectable nanohybrid hydrogel
GA: Glycolic acid
Gel-DSS6: Gelatin methacryloyl modified with bone-targeting peptide
GelMA: Gelatin methacryloyl
GLUT1: Glucose transporter 1
GO: Graphene oxide
GS: Gentamicin sulfate
HA: Hyaluronic acid
HAp: Hydroxyapatite
HCF: Heparin-conjugated fibrin
HIF-1α: Hypoxia-inducible factor-1
HRE: Hypoxia-response element
HUVECs: Human umbilical vein endothelial cells
iPSCs: Induced pluripotent stem cells
LA: Lactic acid
LAP: LAPONITE®
LDHA: Lactate dehydrogenase A
LECs: Lymphatic endothelial cells
LK: Lumbrokinase
Mg^2+^: Magnesium ions
miR-214: MicroRNA-214
MMPs: Matrix metalloproteinases
MSCs: Mesenchymal stem cells
NAMPT: Nicotinamide phosphoribosyltransferase
NIR: Near-infrared
Nha: Nano-hydroxyapatite
OCN: Osteocalcin
PAMg: Polyethylene glycol-alendronate-magnesium
PB: Prussian blue
PCL: Polycaprolactone
PDRN: Polydeoxyribonucleotide
PEG: Polyethylene glycol
PEGDA: Polyethylene glycol diacrylate
Phe: Phenamil
PHD: Prolyl hydroxylase
PKP: Percutaneous kyphoplasty
PLGA: Poly(lactic-*co*-glycolic acid)
PLLA: Poly(l-lactic acid)
PMMA: Polymethyl methacrylate
PVA: Polyvinyl alcohol
PVP: Percutaneous vertebroplasty
QCS: Quaternized chitosan
rBMSCs: Rat bone marrow mesenchymal stem cells
RGD: Arginine-glycine-aspartic acid
rh-BMP-2: Recombinant human bone morphogenetic protein-2
ROS: Reactive oxygen species
RUNX2: Runt-related transcription factor 2
SA: Sodium alginate
SDF-1α: Stromal cell-derived factor 1α
siRNA: Small interfering RNA
SIRT3: Sirtuin 3
SPAAC: Strain-promoted azide–alkyne cycloaddition
SPION: Superparamagnetic iron oxide nanoparticles
3D: Three-dimensional
3D-sEV: 3D-Bioprinted adipose-derived stem cell small extracellular vesicles
3D-SExo: 3D Cultured exosomes modified with superparamagnetic iron oxide nanoparticles
TA: Tannic acid
TAX: Paclitaxel
tFNA: Tetrahedral framework nucleic acid
TGF-β: Transforming growth factor-β
TL: Paclitaxel liposomes
VCM: Vancomycin
VDT: 2-Vinyl-4,6-diamino-1,3,5-triazine
VEGF: Vascular endothelial growth factor

## Author contributions

Xinyue Zhang and Zheng Zhuang: writing – original draft preparation; Xinyue Zhang, Xueyan Zhang and Hao Hu: writing—review and editing; Bo Liu, Mengjia Wu and Chenfei Lu: data curation; Zhan Zhang and Zilu Zhang: validation; Hao Hu: supervision; Yanguo Wang and Hao Hu: project administration; Hao Hu: funding acquisition. All authors have read and agreed to the published version of the manuscript.

## Conflicts of interest

The authors declare no conflicts of interest.

## Data Availability

No primary research results, software or code have been included and no new data were generated or analysed as part of this review.
